# A rich gallery of carbon dots based photoluminescent suspensions and powders derived by citric acid/urea

**DOI:** 10.1038/s41598-021-89984-w

**Published:** 2021-05-18

**Authors:** Joanna D. Stachowska, Andrew Murphy, Claire Mellor, Diogo Fernandes, Ella N. Gibbons, Marta J. Krysmann, Antonios Kelarakis, Engin Burgaz, Joshua Moore, Stephen G. Yeates

**Affiliations:** 1 School of Pharmacy and Biomedical Sciences, University of Central Lancashire, Preston, PR12HE UK; 2 UCLan Research Centre for Smart Materials, School of Natural Sciences, Preston, PR12HE UK; 3 School of Psychology, University of Central Lancashire, Preston, PR12HE UK; 4 Malvern Panalytical, Enigma Business Park, Grovewood Road, Malvern, WR14 1XZ UK; 5 Faculty of Engineering, Department of Metallurgical and Materials Engineering, Ondokuz Mayis University, 55139 Atakum, Samsun, Turkey; 6 School of Chemistry, University of Manchester, Manchester, M13 9PL UK

**Keywords:** Materials science, Nanoscience and technology

## Abstract

In this study we demonstrate simple guidelines to generate a diverse range of fluorescent materials in both liquid and solid state by focusing on the most popular C-dots precursors, i.e. the binary systems of citric acid and urea. The pyrolytic treatment of those precursors combined with standard size separation techniques (dialysis and filtration), leads to four distinct families of photoluminescent materials in which the emissive signal predominantly arises from C-dots with embedded fluorophores, cyanuric acid-rich C-dots, a blend of molecular fluorophores and a mixture of C-dots with unbound molecular fluorophores, respectively. Within each one of those families the chemical composition and the optical properties of their members can be fine-tuned by adjusting the molar ratio of the reactants. Apart from generating a variety of aqueous dispersions, our approach leads to highly fluorescent powders derived from precursors comprising excessive amounts of urea that is consumed for the build-up of the carbogenic cores, the molecular fluorophores and the solid diluent matrix that suppresses self-quenching effects.

## Introduction

A distinct advantage of carbogenic nanoparticles (refereed hereafter as C-dots) is their scalable and low cost synthesis by means of pyrolytic treatment of virtually any type of carbon-rich material, including abundant and renewable resources^1^ such as biomass^2^, grass^3^, leaves^4^, fruits^5^, eggs^6^ or even bacteria^7^. Despite the simplicity and the template-free nature of those bottom-up synthetic approaches, they lead to well-defined C-dots whose structural and morphological characteristics largely depend on the nature of the precursor materials and the synthesis conditions (temperature, time, dispersion medium, etc.)^8–11^. Typically, the radius of pyrolytically derived C-dots falls within 2–20 nm, although nanospheres with size in excess of 50 nm have also been demonstrated^12,13^.

In addition to their preparation ease, C-dots exhibit supreme photoluminescent (PL) properties and show low toxicity for humans and the environment^14^. Owning to this unique combination of desired characteristics, C-dots are systematically explored for the development of photocatalysts^15^, photovoltaics^16^, light emitting diodes^17^, chemical nanosensors^18^, bio-imaging agents^19^, cancer therapy vehicles^20^ and polymer nanocomposites^21,22^. In contrast to the large number of studies centred around their aqueous dispersions, only few reports focus on C-dots nano-powders^23,24^, a nevertheless very interesting class of materials that is prone to self-quenching^25^.

In principle, pyrolytically derived C-dots exhibit excitation wavelength (λ_ex_) dependent PL emission with quantum yields (QY) in the range of 2–15%^2^, while C-dots with improved emission efficiency have also been shown^7,26^. As a general trend, the QY can be substantially improved via heteroatom doping, surface functionalization and passivation^8–10^, while application of a voltammetric field might have a profound impact to the PL intensity^27^. It has been well-established that molecular fluorophores are often coproduced during the synthesis of C-dots^28–30^ and their presence has a profound impact on the optical properties of the resulting material.

In this work we focus on the pyrolytic treatment of citric acid (CA)/urea mixtures (with various molar ratios) that remain the most popular precursor systems for the synthesis of C-dots, an approach that unavoidably proceeds with the parallel formation of molecular fluorophores. We note that a similar approach can be followed using a variety of binary combinations of precursor materials such as urea/malic acid^31^, urea/glycolic acid^31^, urea/sodium citrate^32^, urea/potassium citrate^32^, ethanolamine/CA^28^, (hydroxymethyl)aminomethane/CA^33^, dicyandiamide/CA^34^, etc.

**Figure 1:**
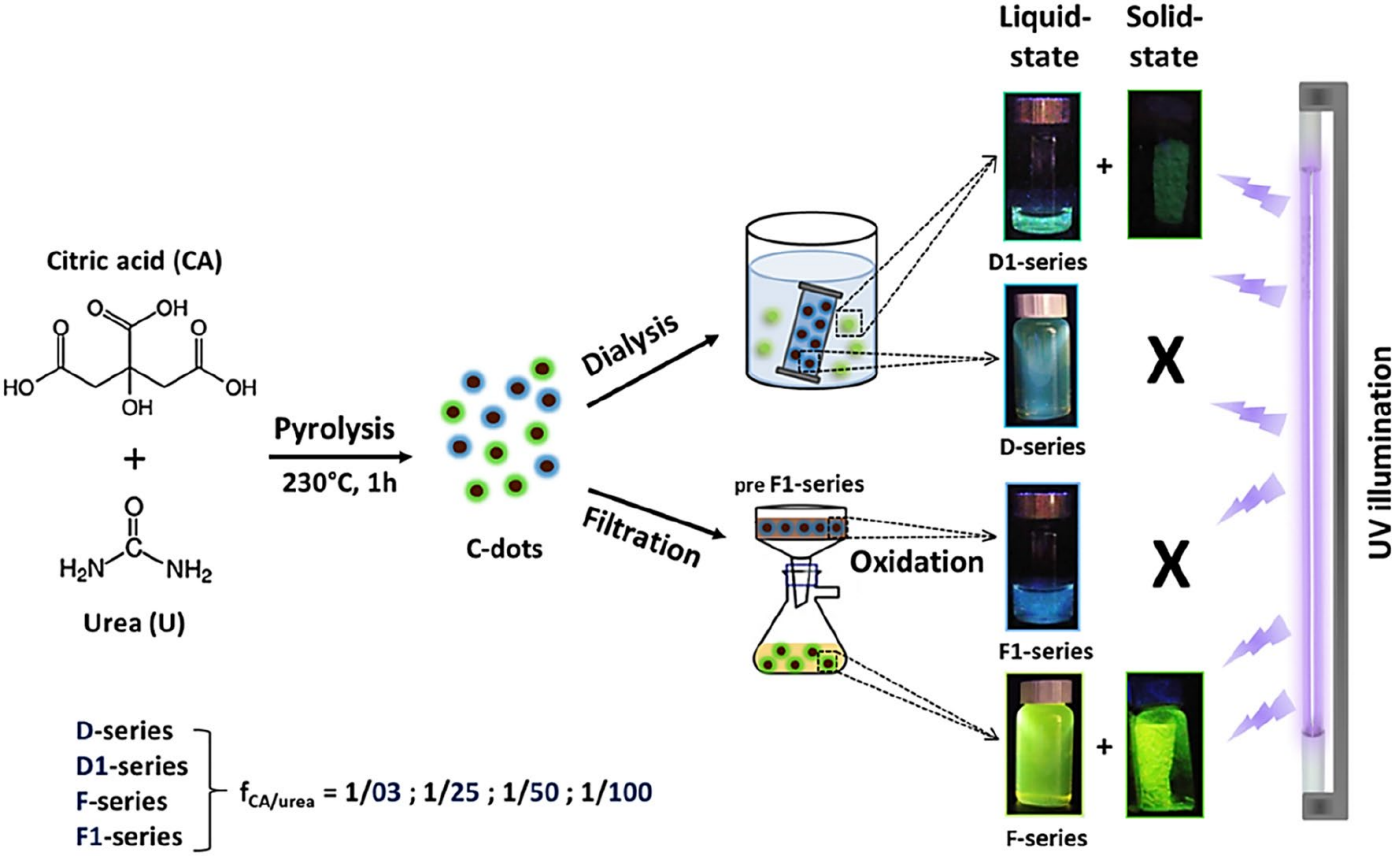
Schematic representation of the synthetic and purification approach followed for the preparation of four series of photoluminescent liquids and solids, all derived by means of pyrolytic treatment of CA and urea precursor mixtures.

Figure 1 outlines the synthetic and purification strategies followed to produce a family of extensively dialysed C-dots (D-series) and the corresponding run-off substances (D1-series). Alternatively, the carbonaceous pyrolysis products were subjected to filtration to generate a family of hybrid materials comprising both nanoparticles and unbound molecular fluorophores (F-series), while the solid residues (preF1-series) were oxidised via HNO_3_ resulting in yet another photoactive type of materials (F1-series).

Within the extensive body of literature centred around the use of CA/urea mixtures as C-dot precursors, the present work distinguishes itself and contributes novel insights in three directions. First, the report further highlights the versatility of the pyrolytic approaches used for the synthesis of C-dots and the rich diversity of the materials produced thereby, via the preparation and characterization of a large gallery of CA/urea derived PL materials. Second, the study allows a direct and meaningful comparison between the D and F series, thus facilitating a quantitative assessment of the effect of molecular fluorophores on the optical properties of C-dots based dispersions. Third, the study discloses effective strategies to generate PL powders by employing excessive amounts of urea to serve simultaneously as the reactant for the synthesis of the molecular fluorophores, the carbon and nitrogen source for the build-up of the carbogenic cores, and as the diluent matrix that suppresses undesired self-quenching effects.

## Results and discussion

### Characterization

#### D-series

**Figure 2:**
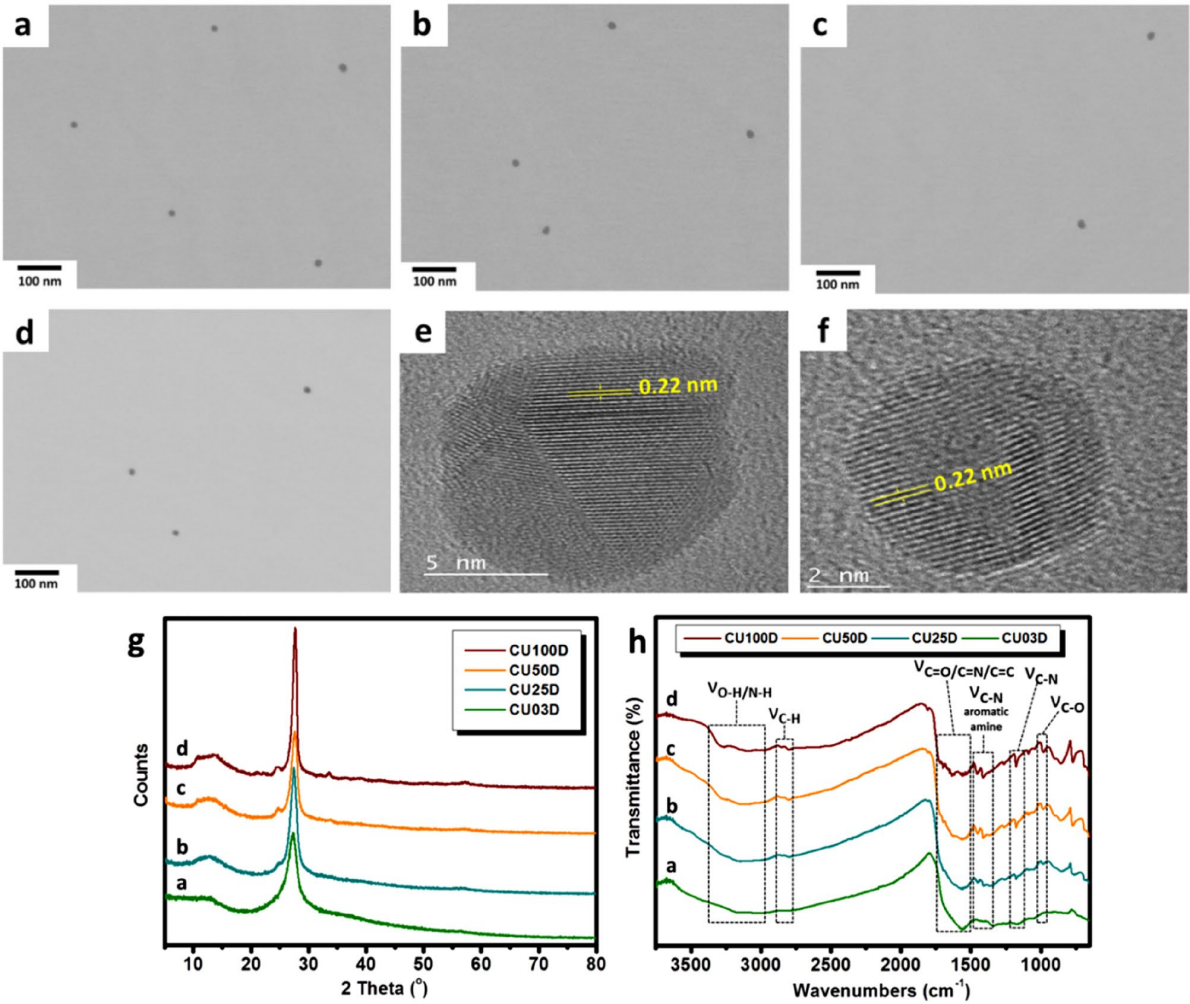
STEM images of CU03D (**a**), CU25D (**b**), CU50D (**c**) CU100D (**d**). HRTEM images of CU03D (**e**) and CU50D (**f**) XRD patterns (**g**) and FTIR spectra (**h**) of D-series.

The Scanning Transmission Electron Microscopy (STEM) images displayed in Fig. 2a–d indicate the spherical nature of CU03D, CU25D, CU50D and CU100D nanoparticles, with mean diameters 14, 16.1, 17.6 and 13.7 nm, respectively (data derived via statistical analysis with N = 200–600 as shown in Fig. S1). Previous studies suggested that the size of C-dots derived from CA/urea mixtures varies from 2 to 16 nm^31,35,36,36–45^. High Resolution Transmission Electron Microscopy (HRTEM) images of CU03D and CU50D (Fig. 2e, f) show lattice spacing close to 0.22 nm that is consistent with (100) facet of graphite. The X-ray diffraction (XRD) patterns of the four members of D-series (Fig. 2g) are dominated by a sharp peak centred at 2θ = 27.6°, in agreement with earlier reports on CA and CA/urea derived C-dots^46–48^. This diffraction peak is close to the (002) lattice spacing of graphite-type materials, further confirming the crystalline nature of the carbogenic core. Elemental analysis (Supplementary Information, Table S1) indicates that the nitrogen content for CU03D, CU25D, CU50D and CU100D, is 21%, 25%, 28% and 33%, respectively and this trend is accompanied by a systematic decrease in the carbon content from 47% for CU03D to 40% for CU100D. At the same time, all members of D-series maintain oxygen content between 24 to 28% and hydrogen content between 3 to 4%. As expected, lower molar ratios of CA to urea (f_CA/urea_) lead to C-dots with enhanced nitrogen content^40,49,50^. For reference it is noted that solvothermal treatment (5 h, 180 °C) of a mixture with f_CA/urea_ = 1:3 results in C-dots containing 39.7% carbon, 23.2% nitrogen, and 32.3% oxygen^51^, while a modified treatment (6 h, 200 °C) of an identical precursor results in C-dots with 45.5% carbon, 31.7% nitrogen, and 21.5% oxygen content^52^.

The full-scan X-ray photoelectron spectroscopy (XPS) survey spectra of D-series (Supplementary Information, Fig. S2) display three characteristic peaks around 286 eV, 400 eV and 532 eV, corresponding to C1s, N1s and O1s, respectively^31,34,39,40,43–45,53^. In addition, deconvolution of the C1s XPS spectra of CU03D,CU25D, CU50D and CU100D (Supplementary Information, Fig. S3) reveals a systematic decrease in the contributions arising from C–C and C=C (284.0 eV) (highest for CU03D) and a corresponding increase in contributions arising from C-N and C=N (286.9 eV) as well as from C-O and C=O (285.4 eV) (lowest for CU03D). Deconvolution of N1s spectra (Supplementary Information, Fig. S4) indicates the dominant population of pyrolytic nitrogen (400.1 eV), while the pyridinic nitrogen (399.1 eV) systematically decreases form 29% for CU03D to 11% for CU100D and the graphitic nitrogen (401.5 eV) increases substantially from 9% for CU03D to 43% for CU100D. Likewise, deconvolution of O1s spectra (Supplementary Information, Fig. S5) indicates that C=O (533.3 eV) increases from 78% for CU03D to 89% for CU100D, while C-O (531.5 eV) decreases from 22% for CU03D to 11% for CU100D. The results from XPS analysis are displayed in Supplementary Information Table S2.

The Fourier Transform Infrared (FTIR) spectra of the four members of D-series (Fig. 2h) display peaks in similar positions attributed to the vibrational modes of=C–H (2810–2197 cm^-1^), C=O or C=N (1653–1556 cm^−1^), C-N from aromatic amine (1462–1340 cm^−1^), C–N (1178 cm^-1^) and C–O (1090–1060 cm^-1^)^48,50,54^. Additionally, the peaks around 1600 cm^−1^ are related to benzene skeleton vibrations^47^ and the broad bands at 3028–3250 cm^−1^ can be assigned to hydrophilic amine (N–H) and hydroxyl (O–H) groups, which ensure good solubility of C-dots in polar solvents^39^.

The zeta potential (ζ) of 5 mg/ml aqueous dispersions of CU03D and CU100D was estimated close to − 57.2 and − 38.2 mV respectively (Supplementary Information, Fig. S6, suggesting the presence of negatively charged surface groups that impart enhanced colloidal stability. In close related systems derived from precursors with f_CA/urea_ = 1:3, ζ has been estimated to be − 40 mV and − 56 mV^55,56^, while for f_CA/urea_ = 1:6 it was found that ζ = − 35.7 mV ^42^.

The Hydrogen-1 Nuclear Magnetic Resonance (^1^H-NMR) spectrum of CU50D (Supplementary Information, Fig. S7) displays two signals at 2.1 ppm and 3.4 ppm, while the corresponding Carbon-13 Nuclear Magnetic Resonance (^13^C-NMR) spectrum (Supplementary Information, Fig. S7) shows one peak at 169.3 ppm (similar peaks were observed for CU03D, CU25D, CU100D). Those spectroscopic signals point to the presence of a heterocyclic compound, presumably CTA (citrazinic acid). Moreover, the peaks at 31.2 ppm and 207 ppm in ^13^C-NMR spectrum refer to sp^3^ and sp^2^ carbon atoms^57^. The Ultraviolet–visible (UV–Vis) spectra of D-series (Supplementary Information, Fig. S7) display an absorption peak at 330 nm and a broader one at 420 nm, indicating the formation of CTA and its supramolecular aggregates, respectively^30,58^. The peak at 270 nm might be attributed to the to the π → π* transitions of the and 4-hydroxy-1H-pyrrolo[3,4-c]pyridine-1,3,6(2H,5H)-trione (HPPT) pyridone ring^30^.

It is well established that the thermal treatment of suitable precursors proceeds with the parallel formation of molecular fluorophores, in a manner that critically depends upon the pyrolysis conditions^28^. In related systems, the generation of fluorophores has been attributed to condensation and ring closure reactions that lead to pyridine-type structures^29^. In a close related study (fCA/urea = 1:10) High-resolution Electrospray Ionisation Mass Spectrometry (HR-LC–ESI–MS) indicated the presence of the blue emissive dye CTA (with UV absorbance peak centred at 330 nm), while High Pressure Chromatography was used to separate a green emissive dye (with UV absorbance peaks centred at 270 nm and 410 nm) that was identified as HPPT by means of Heteronuclear Multiple Bond Coherence^30^.

All in all, the evidence presented above indicates that the members of D-series are composed of spherical C-dots with varying nitrogen content that possess a dense population of surface polar groups. Taken into account that the C-dots have been extensively dialysed against water over a prolonged period of time, it seems reasonable to assume that the organic fluorophores are embedded within the carbogenic structure or, alternatively, are strongly adsorbed on their surface^59^.

#### D1-series

CU50D1 was selected as a representative member of the D1 series for further characterization. The ^1^H-NMR and ^13^C-NMR spectra of CU50D1 (Supplementary Information, Fig. S8) suggest the presence of urea (5.4 ppm in ^1^H-NMR and 160.0 ppm in ^13^C-NMR), biuret (6.8 ppm, 7.1 ppm and 8.5 ppm in ^1^H-NMR and 155.8 ppm in ^13^C-NMR), CYA (cyanuric acid) (150.8 ppm in ^13^C-NMR) and CA (72.9 ppm in ^13^C-NMR). In addition, the peaks seen below 3.5 ppm in ^1^H-NMR and within 25–35 ppm in ^13^C-NMR correspond to the sp^3^ carbons while the peak at 206.9 ppm in ^13^C-NMR spectrum suggest the presence of sp^2^ carbon atoms^32,57^. The UV absorbance spectrum of CU50D1 (Supplementary Information, Fig. S8) indicates the presence of CTA and HPPT molecular fluorophores.

The FTIR spectrum of CU50D1 (Supplementary Information, Fig. S9) shows the vibration peaks of N–H and O–H (3418–3060 cm^-1^), C–H (2810 cm^-1^), C=O or C=N (1655–1571 cm^-1^), C–N from aromatic amine (1412–1346 cm^-1^), C-N (1147 cm^-1^) and C–O (1059 cm^-1^)^48,50,54,60^. Moreover, the vibration peak ca. 1655–1571 cm^-1^ points to C=C stretching, consistent with the presence of benzene skeleton rings^36,47^. In short, CU50D1 is composed of a mixture of CA, CYA, urea, biuret, HPPT and CTA.

#### F-series

In analogy to the D-series, elemental analysis (Supplementary Information, Table S3) indicates that the nitrogen content for CU03F, CU25F, CU50F and CU100F is 31%, 35%, 37% and 38%, respectively and this trend is accompanied by a systematic decrease in carbon content from 36% for CU03F to 29% for CU100F. At the same time within the F-series the oxygen content remains within 28 to 30% and the hydrogen content between 4 and 5%.

The XRD pattern of CU03F (Supplementary Information, Fig. S10) is similar to that seen for CU03D, showing one dominant peak attributed to the (002) lattice spacing of graphite-type structures. However, the XRD patterns of CU25F, CU50F and CU100F (Supplementary Information, Fig. S10) show additional crystalline peaks, with the most pronounced of them centred at 22.2°, 24.6°, 29.3° and 35.4°, corresponding to the (112), (221), (003) and (330) crystal planes of urea.

The ^1^H-NMR spectrum of CU50F (Supplementary Information, Fig. S11) is governed by the peaks at 7.2 and 8.6 ppm stemming from biuret, the peak at 5.6 ppm stemming from urea, while other peaks seen below 3.5 ppm are attributed to the sp^3^ carbons^32^. Likewise, the ^13^C-NMR spectrum of CU50F (Supplementary Information, Fig. S11) is governed by the peaks at 160.2 ppm and 155.9 ppm, stemming from urea and biuret, respectively. The ^13^C-NMR spectrum also displays peaks in the range 25–35 ppm and 150–210 ppm, confirming the presence of both sp^3^ and sp^2^ hybridized carbon atoms^57^, while the additional signal at 151 ppm indicates the presence of CYA^36,61^. The UV spectra of F-series are governed by the peaks at 250, 270, 320, 420 nm, consistent with the presence of CTA and HPPT (Supplementary Information, Fig. S11).

In this work particular emphasis was given to the detection of melamine traces, a possible product of urea’s thermolysis^61^ that is known to impose health risks to humans. To that end, a commercially available melamine test kit was used to confirm that the concentration of melamine in the powder was below 5 ppm. In conclusion, the evidence presented above indicates that each one of the members of F-series represents a multicomponent system comprising C-dots that coexist with HPPT, CTA, urea, CYA and biuret.

#### F1-series

**Figure 3:**
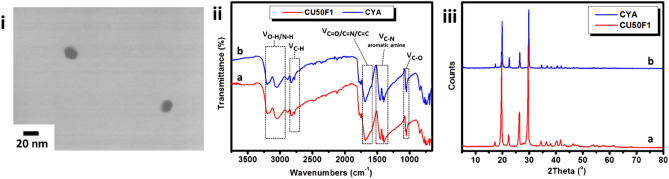
STEM image of CU50F1 (i) as well as FTIR spectra (ii) and XRD patterns (iii) of CU50F1 (**a**) and CYA (**b**).

CU50F1 was selected as a representative member of the F1 series for further characterization. The STEM image shown in Fig. 3i suggests that CU50F1 are spherical nanoparticles with diameter 15 nm. The FTIR spectrum of CU50F1 (Fig. 3ii) close resembles that of the CYA (also displayed in Fig. 3ii for comparison) given that are both dominated by the vibrational peaks at 3199 cm^−1^, 3046 cm^−1^, 1685 cm^−1^, 1452 cm^−1^, 1050 cm^−1^ that are assigned to O–H band of hydroxyl group, N–H band, C=O band as well as C–N band of aromatic amine and C-O, respectively. Note that the oxygen content of CU50F1 is 34% compared to 28% for its preCU50F1 counterpart. Moreover, the ^13^C-NMR spectrum of CU50F1 shows a characteristic peak at 151.1 ppm, corresponding to the main peak seen for CYA (Supplementary Information, Fig. S12). The close matching between the XRD patterns of CU50F1 and CYA (Fig. 3iii) further supports the conclusion that CU50F1 incorporates significant amounts of CYA crystals. A similar system comprising CYA-rich C-dots has been previously prepared via infrared-assisted pyrolysis of a mixture comprising CA and urea with f_CA/urea_ = 1:3^36^.

## Photoluminescence properties in water

**Figure 4:**
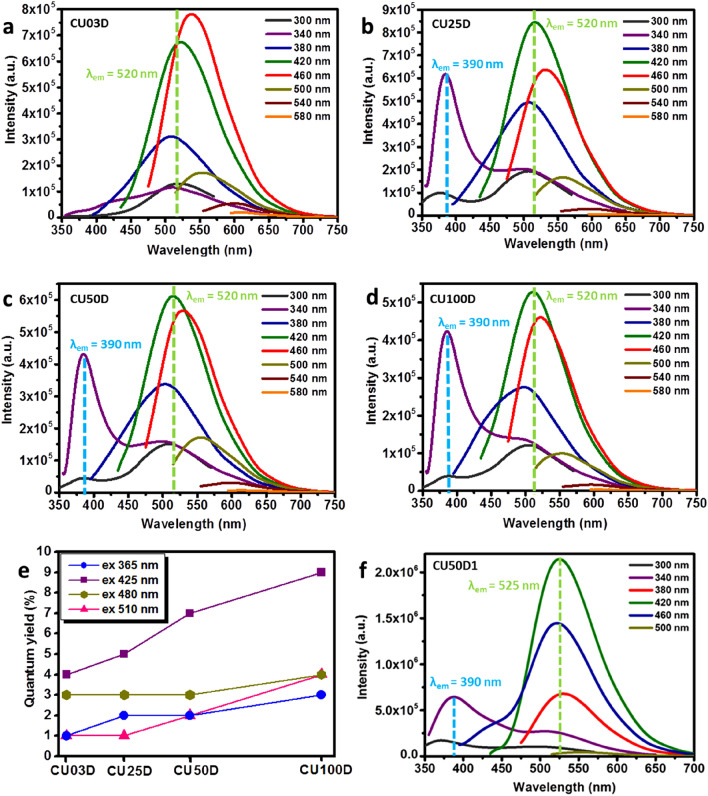
Fluorescence emission spectra (under the various excitation wavelengths indicated) of 0.1 mg/mL aqueous dispersions of CU03D (**a**), CU25D (**b**), CU50D (**c**) and CU100D (**d**), the graph showing the corresponding QY values at λ_ex_ = 365 nm (circles), 425 nm (squares), 480 nm (diamonds), 510 (triangles) (**e**) as well as the fluorescence emission spectra (under the various excitation wavelengths indicated) of 0.1 mg/mL aqueous dispersion of CU50D1 (**f**).

CU03D exhibits excitation wavelength dependent emission characteristic of its carbogenic nature, while for CU25D, CU50D and CU100D contributions stemming from the CTA units (λ_em_ = 390 nm) are also present (Fig. 4a–d). Furthermore, for all samples an emissive signal consistent with HPPT (λ_em_ = 525 nm) can be observed. The maximum PL emission was recorded at λ_ex_ = 460 nm for CU03D, but at λ_ex_ = 420 nm for the other three members of the D-series. In moving form CU03D towards CU100D, a systematic blue shift is observed at λ_ex_ = 460 nm (Supplementary Information, Fig. S13). It has been suggested that even hybridization of frontier orbitals between edge N atoms and graphene quantum dots (GQDs) results in blue-shifting^62^. As shown in Fig. 4e, the QY falls within 1–4% for CU03D, 1–5% for CU25D, 2–7% for CU50D and 2–9% for CU100D, thus exhibiting a systematic enhancement with nitrogen content. As expected, the PL curves of CU50D1 (Fig. 4f) are dominated by two excitation wavelength independent contributions stemming from CTA (λ_em_ = 390 nm) and HPPT (λ_em_ = 525 nm), respectively.

**Figure 5:**
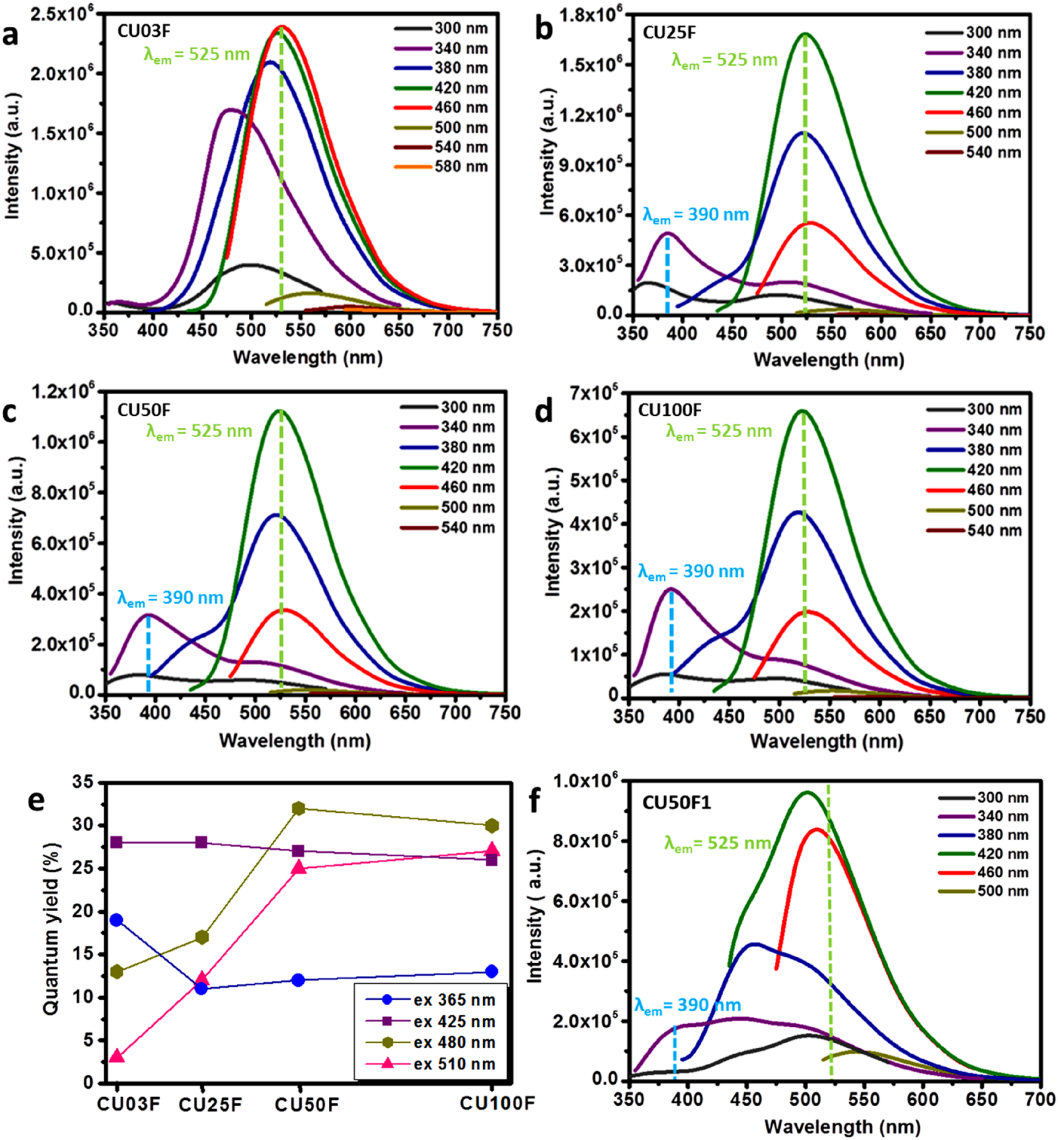
Fluorescence emission spectra (under the various excitation wavelengths indicated) of 0.1 mg/mL aqueous dispersions of CU03F (**a**), CU25F (**b**), CU50F (**c**) and CU100F (**d**), the graph presenting the QY of CU03F, CU25F, CU50F and CU100F at λ_ex_ = 365 nm (circles), 425 nm (squares), 480 nm (diamonds), 510 nm (triangles) (**e**) and fluorescence emission spectra (under the various excitation wavelengths indicated) of 0.1 mg/mL aqueous dispersions of CU50F1 (**f**).

The CTA and HPPT emissive signals are hard to discern for CU03F, but are clearly observed for CU25F, CU50F and CU100F (Fig. 5a–d). All members of F-series also show pronounced excitation wavelength dependent emissions and the QY falls within 3–28% for CU03F, 11–28% for CU25F, 12–32% for CU50F and 13–30% for CU100F (Fig. 5e). Those values are much higher compared to the D-series as a direct consequence of the presence of fluorophores molecules. A survey in the literature suggests that QY of C-dots derived by identical precursor mixtures with f_CA/urea_ = 1:3 vary considerably from 9%, to 27% up to 73% (λ_ex_ = 410 ± 10 nm)^51,57,63,64^.

It is well-understood that such pronounced discrepancies in QY might originate from variations related to the purification/size separation procedures that, in turn, largely determine the amount of fluorophores that are removed away from the system. Our experimental protocol allows a quantitative assessment of the impact of molecular fluorophores on the emission of C-dots based dispersions, in the sense that a comparison between D and F series indicates that the presence of unbound molecular fluorophores improves the QY by a factor between three to five. We note that all members of F-series apart form C-dots and organic fluorophores, also contain urea, biuret and citric acid that do not affect the PL intensity and CYA that is known to quench the PL of C-dots.

**Figure 6:**
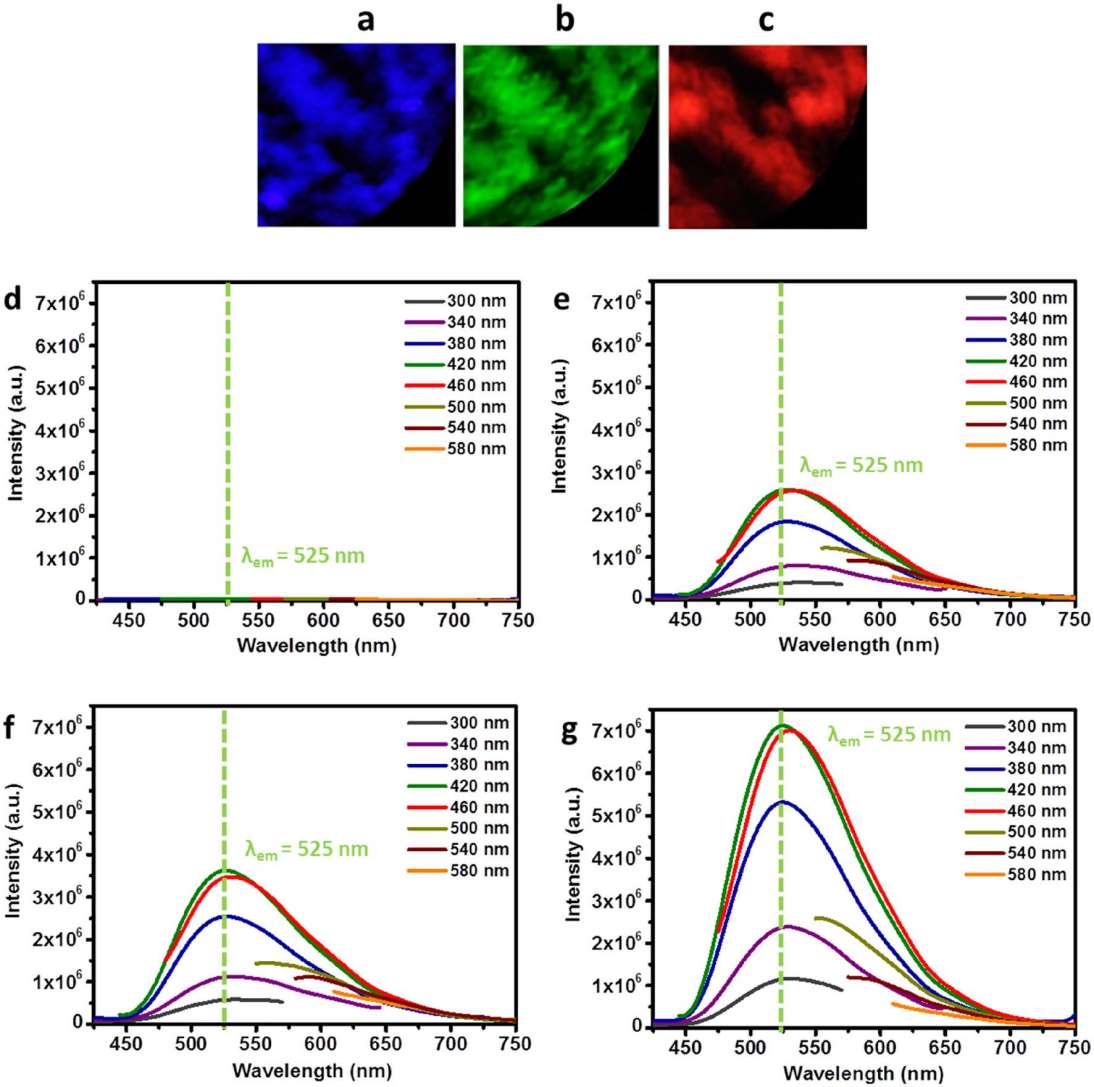
Fluorescent microscopy images of CU100F nanopowders on a glass slide illuminated using a violet (**a**), blue (**b**) and green (**c**) excitation wavelength and the fluorescence emission spectra (under the various excitation wavelengths indicated) of CU03F (**d**), CU25F (**e**), CU50F (**f**) and CU100F (**g**) nanopowders.

Interestingly, CU50F1 exhibits excitation wavelength dependent PL emission (Fig. 5f) with QY equal to 10, 32, 10 and 3% at λ_ex_ = 365, 425, 480, 510 nm, respectively. For reference, the CYA-enriched C-dots previously reported exhibit QY between 8 to 16% (λ_ex_ = 360 nm), displaying asymmetric lamps at 450 and 520 nm^36^ that close resemble those seen in Fig. 6f. In principle, with respect to the PL mechanism of C-dots, three emissive modes have been identified originating from surface defects, the presence of isolated sp^2^ islands with sp^3^ domains and crosslink-enhanced emissions, respectively^65,66^.

The average fluorescence lifetime (τ_avg_) at λ_ex_ = 375 and 450 nm was found to be between 7.5 ns to 9.2 ns for D-series (Supplementary Information, Fig. S14) and between 5.6 ns to 7.0 ns for the F-series (Supplementary Information, Fig. S15). A previous study reported that C-dots derived via pyrolytic treatment (1 h, 250 °C) of CA/urea mixture with f_CA/urea_ = 1:3 shows τ_avg_ = 6.7 ns, while hydrothermal treatment of the same precursor mixture resulted in the generation of N-GQDs with τ_avg_ = 8.1 ns^31,67^. At the same time, a single exponential PL decay with lifetime of 6.4 ns has been demonstrated for CTA^68^, while C-dots are known to exhibit multi-exponential delay stemming from heterogeneous emitting states in a single C-dot or within a larger C-dot ensemble^69^.

## Nanopowders

All members of D-series as well as CU03F are not photoactive in their solid state, presumably due to pronounced self-quenching effects. In contrast, CU25F, CU50F and CU100F nanopowders show colour-tuneable characteristics in the sense that they adopt blue, green and red colour when illuminated by violet, blue and green radiation, respectively (Fig. 6a–c). The fluorescence behavior of F- powders originates from the presence of C-dots, given that fluorescent microscopy images of urea/ CA precursor powders do not show any fluorescence, while the D1-powders show only blue fluorescence under identical conditions. The emissive peaks of those nanopowders are broad (in line with the behaviour previously reported^74^) and their intensity systematically increases with the nitrogen content as shown in Fig. 6d–g.

The PL lifetime decay of the powders at λ_ex_ = 375 nm can be fitted by a bimodal function, showing τ_avg_ close to 5.85 ns for CU25F, 5.95 ns for CU50F and 6.45 ns for CU100F, while similar trends were observed at λ_ex_ = 450 nm (Supplementary Information, Fig. S16).

Effective strategies to generate fluorescent C-dots based nanopowders rely on the use of a suitable diluent powder^23^, the engineering of a carbogenic coating on the surface of nanoparticles^70^ or microspheres^71^, the presence of long aliphatic chains on the surface of the C-dots^72^ and effects related to aggregation-induced emission^73^. A previous study discloses PL nanopowders with decreased sp^2^ domains derived pyrolytically from mixtures with f_CA/urea_ = 1:26; however this strategy depends on a rather tedious purification protocol by means of grinding to fine powder, dispersion in ethanol, centrifugation, dispersion in water, dialysis, filtration and freeze-drying^74^.

In this work we demonstrate a facile approach to generate PL nanopowders by employing precursor systems comprising extreme excess of urea, followed by a single filtration step as the sole size separation process. The role of urea is triple: it serves as the building block of the carbogenic core, as a reactant for the synthesis of molecular fluorophores, while its thermolysis products and its unreacted fraction are used as the diluent materials that eliminate undesired self-quenching effects. In that sense our strategy demonstrates a cost effective pathway towards the production of functional materials for highly demanding applications.

For convenience, Table 1 summarises the composition and performance characteristics of the four families of PL materials synthesised in this study.

**Table 1:** Composition and PL behaviour of D-, D1-, F-, F1- series.

Type of material	Composition	Liquid state, QY	Solid state PL
D-series	C-dots, CTA and HPPT entrapped in C-dots	CU03D, 1–4%	No
		CU50D, 2–7%	No
		CU25D, 1–5%	No
		CU100D, 3–9%	No
D1-series	CYA, CA, urea, biuret, HPPT	Yes	Yes
F-series	C-dots, CYA, CTA, urea, biuret, HPPT	CU03F, 3–19%	No
		CU25F, 11–28%	Yes
		CU50F, 12–32%	Yes
		CU100F, 13–30%	Yes
F1-series	CYA-rich C-dots	CU50F1, 3–32%	No

## Toxicity/Bioimaging

**Figure 7:**
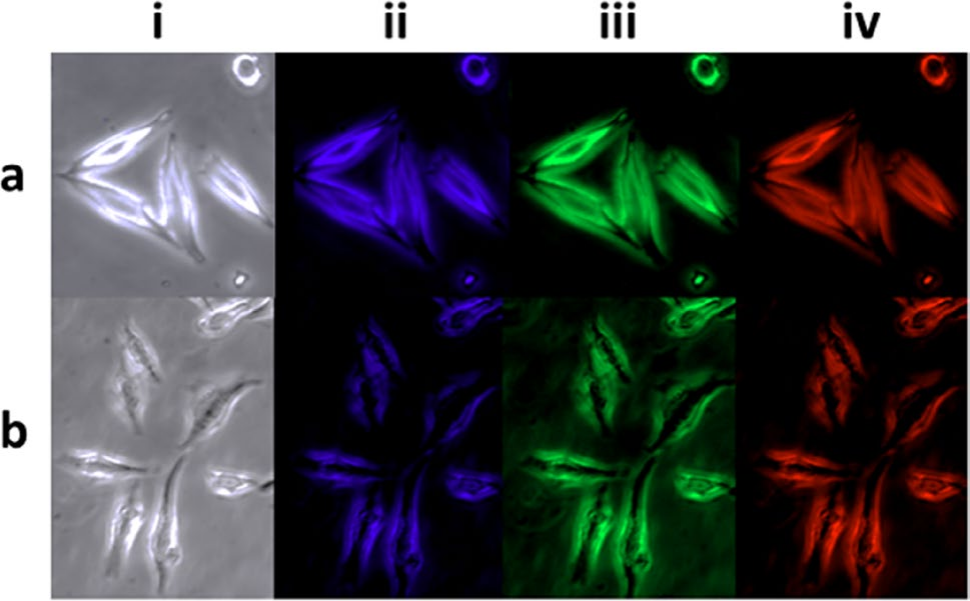
Fluorescent microscope images of HeLa cell line incubated with 100 μg/mL of CU100D (**a**) and CU100F (**b**) under the bright field (i) as well as the UV (ii), blue (iii) and green (iv) excitation wavelengths.

The toxicity of D-series and F-series was evaluated with respect to HeLa cells using standard MTT (3-(4,5-dimethylthiazolyl-2)-2,5-diphenyltetrazolium bromide) assays. The results indicated that the mortality of HeLa cells incubated for 24 h with C-dots (at concentrations up to 1 mg/mL) was lower that 10% (Supplementary Information, Fig. S17). Suffice it to say that such low toxicity values, rarely seen for other classes of photoactive materials, is highly desirable for biomedical applications, although experimental evidence suggests that the surface functional groups might have a pronounced effect on the cytotoxicity of C-dots^52,63^. Moreover, fluorescent images (Fig. 7) indicated that upon incubation, C-dots become internalised by HeLa cells, illuminating both the cytoplasm and the cells membranes. Those promising results indicate that both D and F series are ideal candidates for multicolour imaging and cell labelling given that they emit strong PL signals under a wide range of excitation wavelengths.

The F-series shows excellent antimicrobial properties against *Escherichia coli* (causing reduction in bacterial colonies between 99.5% and 99.9%) and *Staphylococcus aureus* strain (causing reduction in bacterial colonies between 99.2% and 99.9%) (Supplementary Information, Table S4). Likewise, the D-series exhibits significant antimicrobial properties against *E. coli* and *S. aureus* that is more pronounced for CU03D, CU25D, CU50D, but falls to 79% against *E. coli* for CU100D. Note that derivatives of CTA and molecules structurally similar to HPPT as well as CA are known to exhibit significant antimicrobial properties^75–77^.

## Conclusions

In conclusion, we provide an easy-to-follow roadmap to synthesise a large gallery of photoactive materials derived via the thermal treatment of CA and urea, by varying the molar ratio of the components in the precursor mixture and by employing two standard size separation strategies, i.e. dialysis and filtration. In the latter case we report the preparation of PL nanoparticles derived from both the filtrate and the residue, thus suggesting an eco-friendly synthetic approach with minimal waste. Apart from demonstrating the preparation of four families of highly fluorescent aqueous dispersions, we also present the synthesis of colour-tuneable, photoluminescent nanopowders that are derived pyrolytically from mixtures containing extreme excess of urea, followed by a single filtration step. Significantly, the powders that have been subjected to extensive purification (a rather tedious and time consuming procedure) do not exhibit fluorescence emission, while the powders processed by simple filtration (a simple and fast procedure) are ideal candidates for applications related to solid state emission such as fingerprint powders, light emitting diodes, etc. Compared to previous reports, our approach represents by far the most cost and time effective synthetic route for the preparation of highly PL nanopowders based on C-dots.

## Methods

### Materials

Urea (purity 99.0–100.5%) and citric acid (CA) monohydrate (purity 99.5%) were purchased from Sigma Aldrich and Alfa Aesar, respectively. Nitric acid (HNO_3_) 70% and water (purity ≥ 99.9) were bought from Fischer Chemical. The cellulose grade one paper filters (11 µm particle retention) were supplied by Whatman (GE Healthcare Life Sciences). The SnakeSkin Dialysis Tubing membrane 3.5 kDa molecular weight cut-off (MWCO, 35 mm) was purchased from Thermo Fisher Scientific.

### Preparation of D-series, D1-series, F-series and F1-series

The CA monohydrate was mixed with urea and the mixture was placed into a furnace programmed to rise to 230 °C at a rate of 1 °C/min and was then left at 230 °C for 1 h. Four different molar ratios of CA to urea f_CA/urea_ equal to 1:3, 1:25, 1:50 and 1:100 were used to form the products CU03, CU25, CU50 and CU100, respectively. The clustered solid materials received were ground to fine powders using a mortar and pestle. The ground material was dispersed in water and was extensively dialysed against water for 10 weeks using SnakeSkin membrane with 3.5 kDa MWCO to generate the D-series (CU03D, CU25D, CU50D and CU100D). The run-off substances during the first month were collected to form the D1 series (CU03D1, CU25D1, CU50D1 and CU100D1). Alternatively, the pyrolysis products were dispersed in water and passed through cellulose grade one paper filters (11 µm particle retention) to give rise to the F-series (CU03F, CU25F, CU50F and CU100F). The residues on the filters (the preF1-series, comprising preCU03F1, preCU25F1, preCU50F1 and preCU100F1) were oxidised with 3 M nitric acid for 18 h, giving rise to the F1-series (CU03F1, CU25F1, CU50F1 and CU100F1). All samples were freeze-dried and stored in a dry environment before use.

### Characterization

* CHNS Elemental Analyzer* (Flash 2000) equipped with a column for oxygen determination was calibrated against 2,5-(Bis(5-tert-butyl-2-benzo-oxazol-2-yl)thiophene (Thermo Scientific, UK). The C, H, N analysis was carried out in aluminium pans, while oxygen analysis in silver pans (both types of pans were received from CE Instruments). The ^1^*H and *^*13*^*C NMR spectra* were recorded using Bruker Advance-III spectrometer operating at 300 MHz and 75 MHz, respectively by using tetramethylsilane (TMS) as an internal standard. All samples were dissolved in deuterated dimethyl sulfoxide (d-DMSO, Cambridge Isotopes Laboratories Inc.) and scanned 128 times (^1^H-NMR) or 1024 times (^13^C-NMR) at ambient temperature, using a 5 mm NMR tube (Wilmad LabGlass, USA). For comparison the NMR spectra of biuret (Alfa Aesar), CYA (Sigma Aldrich), CTA (Sigma Aldrich), CA (Alfa Aesar) and urea (Sigma Aldrich) were also collected. *FTIR* spectra were recorded by means of ThermoFisher Scientific Ltd Nicolet IS5 spectrometer. Samples were scanned 128 times at a resolution of 2 cm^-1^, within the range of 3750–650 cm^-1^. *XRD patterns* were obtained using a Bruker D2 Phaser equipped with a Lynxeye 1-dimensional detector. The wavelength of the Cu Kα radiation was 0.154 nm. Samples were scanned for 30 min, within the 2θ range from 5° to 80°. *Unisensor Melamine Sensor kit* was used for the detection of melamine. To that end 200 µl of 5 wt% aqueous dispersion of the powders were transferred to a microwell containing gold nanoparticles functionalised with antibody showing high affinity for melamine. A colour development (pink line) within the test zone of a dipstick comprising capture zones, is the signature of a melamine-free sample. The test sensitivity for aqueous solutions is 250 ppb. *Zeta Potential (ζ) measurements* were made using a Zetasizer Ultra (Malvern Panalytical, Ltd) which includes a 10 mW He–Ne laser operating at λ = 633 nm. Dust-free solutions were obtained by filtration through Nylon membrane filters with a pore size of (0.45 μm) (GE Nylon Syringe Filter). The concentration of the dispersions was 5 mg/ml and the temperature was set to 25 °C. The electrophoretic mobility was converted into Zeta Potential assuming Smoluchowski approximation. *Fluorescence microscopy images* were obtained using a Zeiss Axio Scope A1 microscope equipped with band-pass filters. Three excitation wavelengths were used 350 (ultraviolet), 395 (blue) and 590 (green) nm. *STEM experiments* were performed by using JEOL-JSM-7001F. The STEM detector, high vacuum mode and 20–30 kV accelerating voltages were used during experiments. Samples for STEM experiments were prepared by drying a drop of aqueous solutions with concentrations of about 1 mg/mL on carbon-coated copper grids at room temperature. The nanoparticle sizes were analyzed by means of image analysis software ImageJ (Version 1.52a, National Institute of Health, USA). *HRTEM* experiments were performed by using a JEOL JEM-ARM 200CFEG UHR-TEM (equipped with STEM, Cs corrected STEM, EDS, Gatan Quantum GIF and Digital CCD Camera). *Ultraviolet–visible (UV–Vis) spectra* of aqueous dispersion were recorded at room temperature using the UV-3600 spectrophotometer (SHIMADZU,USA)**.** Diluted aqueous solutions with concentration 0.1 mg/mL were placed in Hellma Analytics quartz cuvette (1.0 cm pathlength). XPS analysis was carried out using ESCA2SR spectrometer (Scienta Omicron GmbH) equipped with a monochromatic Al Kα X-ray radiation source. (1486 eV) with 15 mA emission at 300 W. Any differential charging effects were removed using a charge neutralising low-energy electron gun (FS40A, PREVAC) under X-ray illumination. High resolution core level scans were measured with a pass energy of 20 eV, and overview surveys with 80 eV pass energy. Gaussian–Lorentzian peak shapes were used in CASAXPS for spectral deconvolution. *Fluorescence spectroscopy and QY.* Fluorescence spectra of solutions and nanopowders were recorded at room temperature using a Horiba Fluoromax spectrofluorometer and the samples were excited at wavelengths between 320–500 nm, with 30 nm increment. For the solid state characterization, the sample holder was placed at 30 degree angle with respect to the incident beam. The relative *QY* of C-dots solutions were calculated using the Equation :

1 $${\text{QY}} = {\text{QY}}_{{\text{R}}} \times \left( {\frac{{\text{m}}}{{{\text{m}}_{{\text{R}}} }}} \right) \times \left( {\frac{{{\upeta }^{2} }}{{{\upeta }_{{\text{R}}}^{2} }}} \right)$$

where $QY_{R}$ is the QY of the reference dye, m and ${\text{m}}_{{\text{R}}}$ refer to the slope calculated from a linear regression of the examined material and the reference dye,$\eta^{2}$ and $\eta_{R}^{2}$ are the refractive indexes of the solvents used for the examined material and the reference dye, respectively. The anthracene (QY = 0.27), fluorescein (QY = 0.79), rhodamine 6G (QY = 0.95) and rhodamine B (QY = 0.70) were dissolved in ethanol and used as standard reference dyes with excitation wavelengths 365 nm, 425 nm, 480 nm and 510 nm, respectively. Anthracene was purchased from Sigma Aldrich while the other dyes were received from Alfa Aesar). All the measurements of absorbance and integrated PL intensity were conducted using a Hellma Analytics quartz cuvette with 1.0 cm pathlength. Additionally, the absorbance values were kept below 0.10, in order to minimize re-absorption effects.

### Fluorescence lifetimes

Fluorescence lifetime decays were recorded using Edinburgh Instruments LifeSpec-II equipped with two high-repetition rate picosecond pulsed diode lasers (EPL-375 and EPL-450) operating at 375 nm and 450 nm, respectively. The detector signals were processed using Time-correlated Single Photon Counting (TCSPC) data acquisition unit. The PL lifetime decays for aqueous dispersions were collected with 10,000 peak counts in quartz cuvette (10 mm path length) at room temperature over 200 ns time range. The PL lifetime decays of solid powders were collected in quartz slides (0.5 mm pathlength) with the same parameters. The instrument response function (IRF) was recorded at the same experimental parameters, using a dilute aqueous dispersion of colloidal silica (Ludox HS-30) for the solutions and empty quartz slides for the powders. The average PL lifetimes (τ_avg_) were calculated from the Equation:

2 $$\tau_{{{\text{avg}}}} = \frac{{\sum \alpha_{i} \tau_{i}^{2} }}{{\Sigma \alpha_{i} \tau_{i} }}$$

where $\tau_{i}$ is the time component of multiexponential decay fitting and $\alpha_{i}$ is the fractional weight for each time component).

*Cytotoxicity studies* were assessed with the use of the standard MTT (3-(4,5-dimethylthiazolyl-2)-2,5-diphenyltetrazolium bromide) assay (Sigma Aldrich) and the epithetical HeLa cervix cancer cell line. This immortalised cell line is very well characterised with its full genome being sequenced ^78^ and is considered as a model line by regulatory odies such as the OECD, being widely used for the assessment of nanoparticles toxicity^79,80^. The HeLa cells were cultured in Dulbecco's Modified Eagle Medium (DMEM, ThermoFisher Scientific) supplemented with 10% Fetal Bovine Serum (FBS, ThermoFisher Scientific) and 1% Penicilin-Streptomycin (10,000 U/mL, ThermoFisher Scientific). Initially, 50 μL of cell suspensions with the density 5 × 10^4^ cells/mL were seeded in a 96-well plate and left in the incubator (37 °C, 5% CO_2_) overnight to promote adhesion of the cells to the bottom of each well. After 24 h, the cells were incubated with various concentrations of C-dots solutions (5–1000 μg/mL in DMEM) for 24 h. Subsequently, 20 μL of MTT solution (5 mg/mL) was added into each well and left for 2 h of incubation. In the last step, 150 μL of lysis buffer (0.7 M) was added into each well to dissolve formazan crystals and incubated for further 3 h. Finally, the optical density (OD) of each sample was recorded using a microplate reader (ThermoFisher Scientific, UK) at a wavelength 595 nm. The cell viability was calculated based on the Equation:

3 $${\text{Cell}}\;{\text{viability}} \left[ \% \right] = \frac{{{\text{OD }}\;{\text{treated}}}}{{{\text{OD}}\;{\text{control}}}} \times 100$$

where OD_treated_ and OD_control_ were obtained in the presence and the absence of C-dots, respectively. All assays were performed at least three times and an average value was calculated). In vitro* fluorescence imaging.* The HeLa cells cultured in DMEM supplemented with 10% FBS (v/v) and 1% Penicilin-Streptomycin (v/v) were seeded in 6-well plate and left overnight in the incubator at 37 °C (5% CO_2_). Afterwards, 100 μg/mL C-dots solutions were added to each well with the HeLa cells and incubated for further 24 h. Then, the media was removed and the cells were washed three times with phosphate buffer saline (PBS) buffer (pH 7.2) to remove not internalized C-dots. The fluorescence imaging in live cells was performed under bright field as well as with λ_ex_ = 366 nm, 488 nm and 512 nm. *Antimicrobial studies. A. Culturing Method:* The method has been described in detail elsewhere^81^. In brief, nutrient broth was inoculated with a single loop of bacteria and incubated at 37 °C, for 24 h at 200 rpm in a SciQuip Incu-Shake MIDI orbital shaker (SciQuip Ltd, Newtown,Wem, Shropshire, UK). Subsequently, the bacteria cultures were centrifuged twice, the supernatant was discarded, and the resuspended cultures were diluted in nutrient broth to obtain an absorbance reading equivalent to a 0.5 McFarlane standard as recorded by Biochrom WPA S800 visible spectrophotometer*. B. Antimicrobial testing method:* 0.1 g ± 0.01 g of C-dots was dispersed in 1 ml nutrient broth and the dispersion was autoclaved. Subsequently, 8 ml sterile nutrient broth and 1 mL of strain were added to each dispersion and placed into a shaking incubator set to 200 rpm at 37 °C. After 24 h of incubation, 1 mL aliquot was removed from each suspension and was serially diluted by adding 100µL of it into 900µL ¼ strength Ringer’s solution. This allowed an order of magnitude dilution at a time and this process was repeated several times so that the number of colonies would fall within the range 30–300. To that end, 100µL of each one of the series of the diluted suspensions was spread onto nutrient agar plates using a sterile plastic spreader. Plates were incubated for 24 h at 37 °C, then the number of colonies was determined. The percentage decrease in comparison to the control plate was calculated using the Equation:

4 $${\text{Bacterial}}\,{\text{colony}}\,{\text{decrease }}\left[ \% \right] = \frac{{{\text{Control}}\, - \,{\text{Test}}}}{{{\text{Control}}}} \times 100$$

where Control and Test stands for the number of colonies in the plates in the absence and in the presence of C-dots, respectively under otherwise identical conditions.

For each type of C-dots the experiments were conducted in triplicates and the average values were determined.

## Supplementary Information

Supplementary Information.
