# Supplementary material for: A rich gallery of carbon dots based photoluminescent suspensions and powders derived by citric acid/urea
Source: Sci Rep. 2021 May 18;11:10554. doi: 10.1038/s41598-021-89984-w (PMC8131706; doi:10.1038/s41598-021-89984-w)
Supplement: Supplementary file 1 — Supplementary Information. [file 41598_2021_89984_MOESM1_ESM.pdf]

A rich gallery of carbon dots based photoluminescent suspensions and powders derived by citric acid/urea

Joanna D. Stachowska[1], Andrew Murphy[2], Claire Mellor[3], Diogo Fernandes[4], Ella N. Gibbons[1], Marta J. Krysmann[1], Antonios Kelarakis[2]*, Engin Burgaz[5], Joshua Moore[6] and Stephen G. Yeates[6]

[1]University of Central Lancashire, School of Pharmacy and Biomedical Sciences, Preston PR12HE, UK
[2]UCLan Research Centre for Smart Materials, School of Natural Sciences, Preston PR12HE, UK
[3]University of Central Lancashire, School of Psychology, Preston PR12HE, UK
[4]Malvern Panalytical, Enigma Business Park, Grovewood Road, Malvern WR14 1XZ, UK
[5]Ondokuz Mayis University, Faculty of Engineering, Department of Metallurgical and Materials Engineering, 55139 Atakum, Samsun, Turkey
[6]University of Manchester, School of Chemistry, Manchester M13 9PL, UK
* akelarakis@uclan.ac.uk

Table S1. Elemental analysis of D-series

Material	D-series			
	C	H	N	O
CU03D	47%	4%	21%	28%
CU25D	45%	4%	25%	26%
CU50D	44%	3%	28%	25%
CU100D	40%	3%	33%	24%

Table S2. XPS analysis for CU03D, CU25D, CU50D and CU100D.

Material	C1s				O1s		N1s		
	C-C<br>C=C	C-O<br>C-N	C=O<br>C=N	O=C-O	C=O	C-O	Graphitic-N	Pyrrolic-N	Pyridinic-N
CU03D	51%	18%	25%	6%	78%	22%	9%	62%	29%
CU25D	45%	18%	30%	7%	85%	15%	18%	61%	21%
CU50D	44%	19%	31%	6%	87%	13%	25%	59%	16%
CU100D	34%	21%	38%	7%	89%	11%	43%	46%	11%

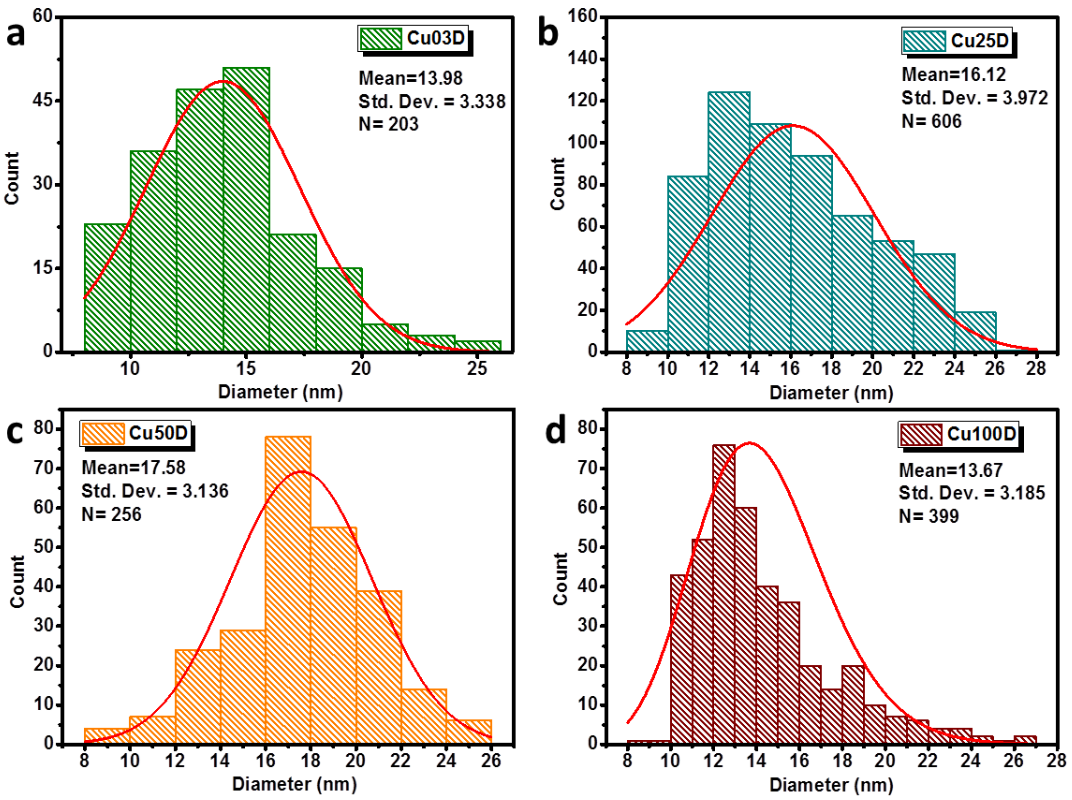

Figure S1. Size distribution of CU03D (a), CU25D (b), CU50D (c), CU100D (d). The red line is the Gaussian fitting curve.

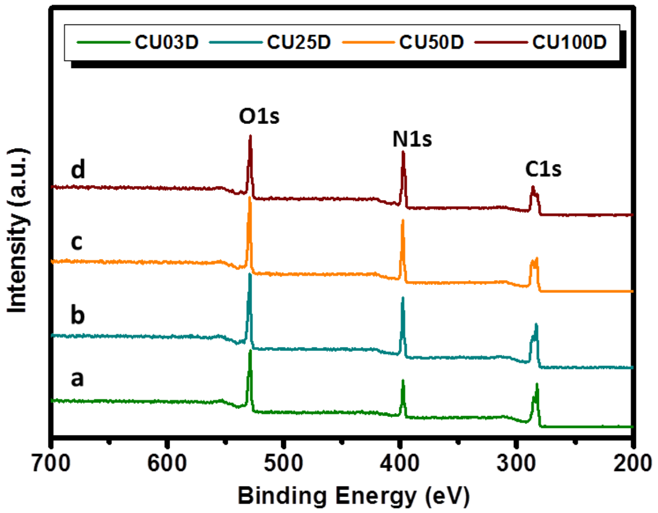

Figure S2. The full-scan XPS survey spectra of CU03D (a), CU25D (b), CU50D (c) and CU100D (d).

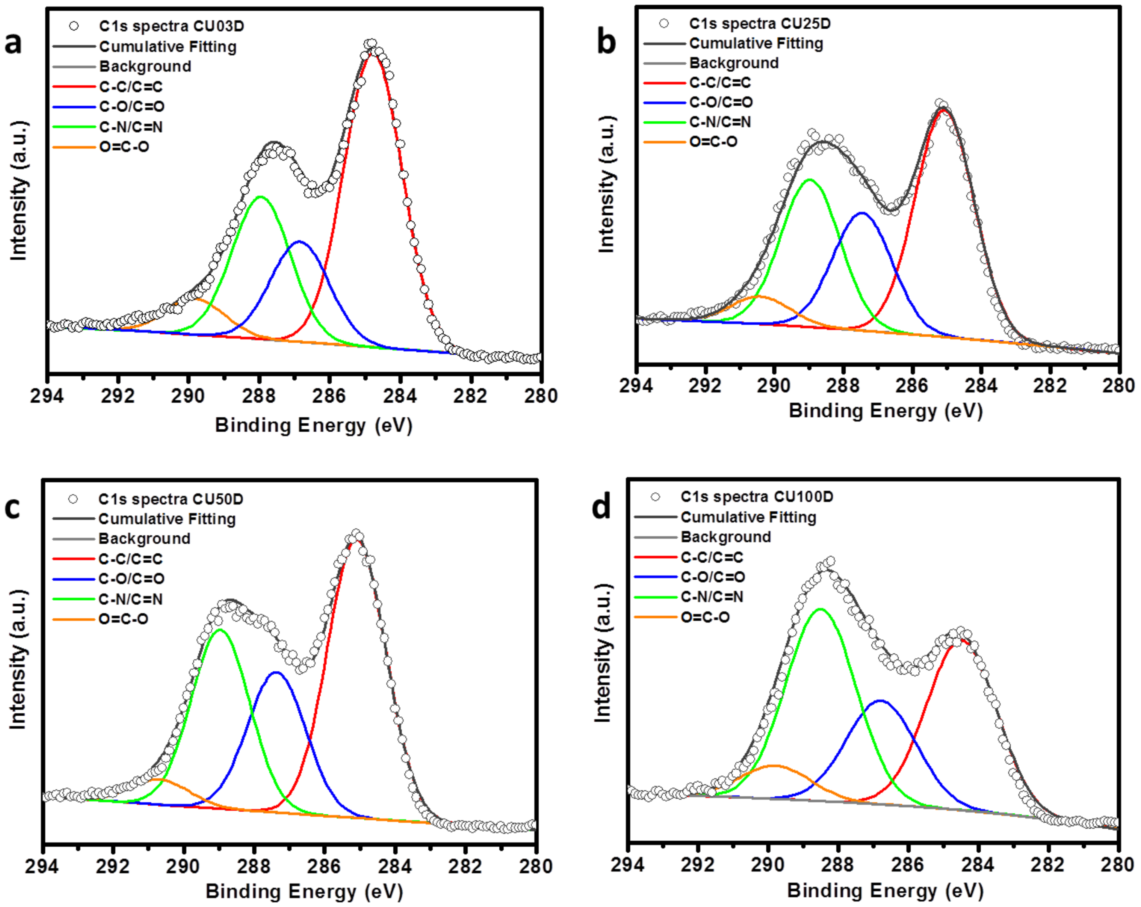

Figure S3. Deconvolution of C1s XPS spectra of CU03D (a), CU25D (b), CU50D (c) and CU100D (d). The points represent the experimental data and the lines correspond to the fitted curves.

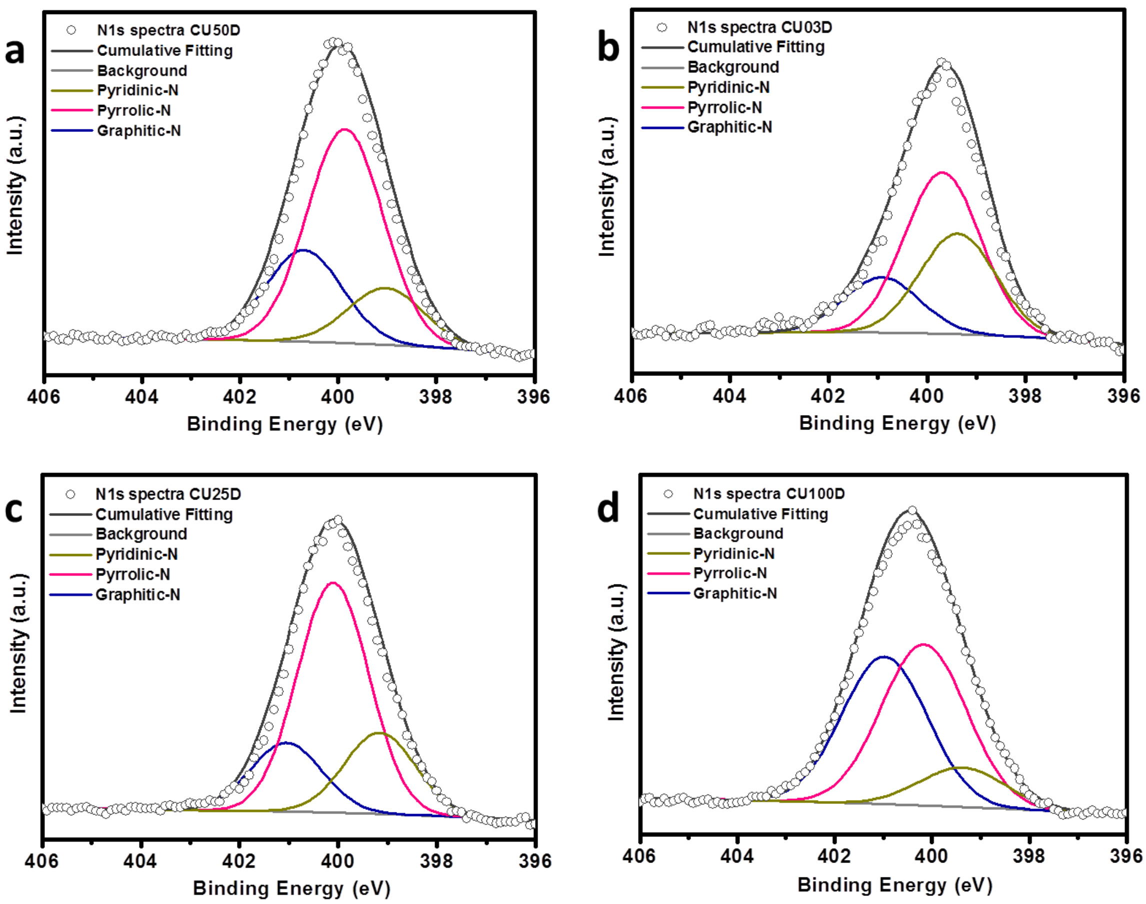

Figure S4. Deconvolution of N1s XPS spectra of CU03D (a), CU25D (b), CU50D (c) and CU100D (d). The points represent the experimental data and the lines correspond to the fitted curves.

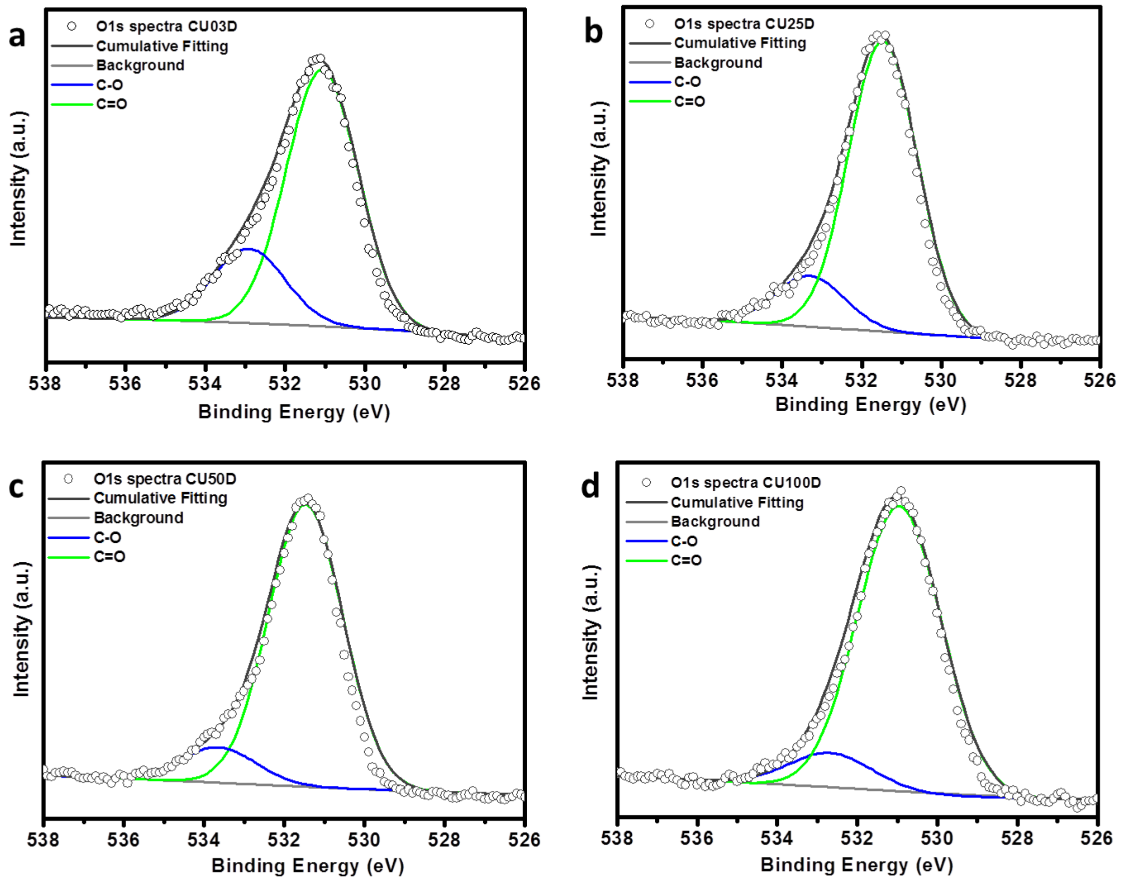

Figure S5. Deconvolution of O1s XPS spectra of CU03D (a), CU25D (b), CU50D (c) and CU100D (d). The points represent the experimental data and the lines correspond to the fitted curves.

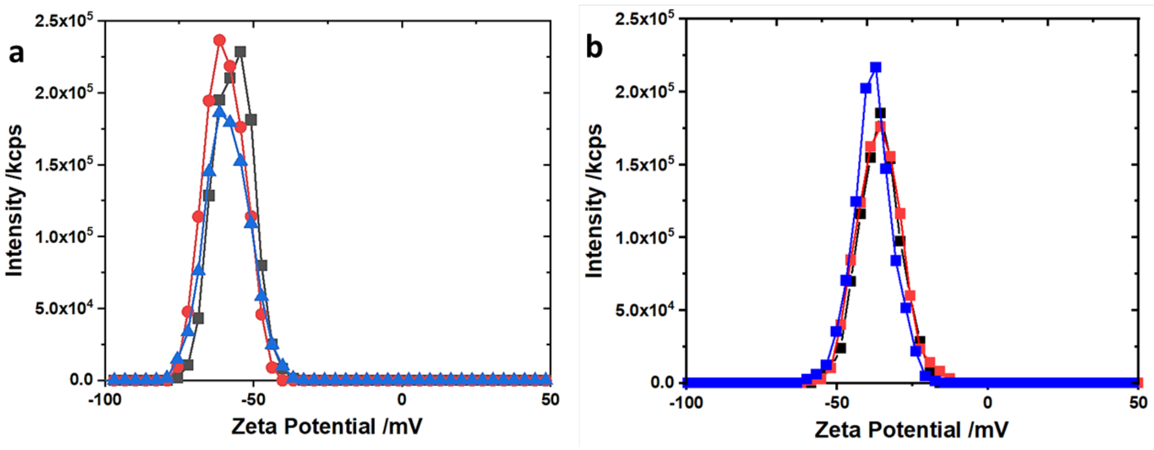

Figure S6. Zeta potential (ζ) of 5 mg/ml aqueous dispersion of CU03D (a) and CU100D (b). For each dispersion three independent measurements are displayed and the average value was calculated

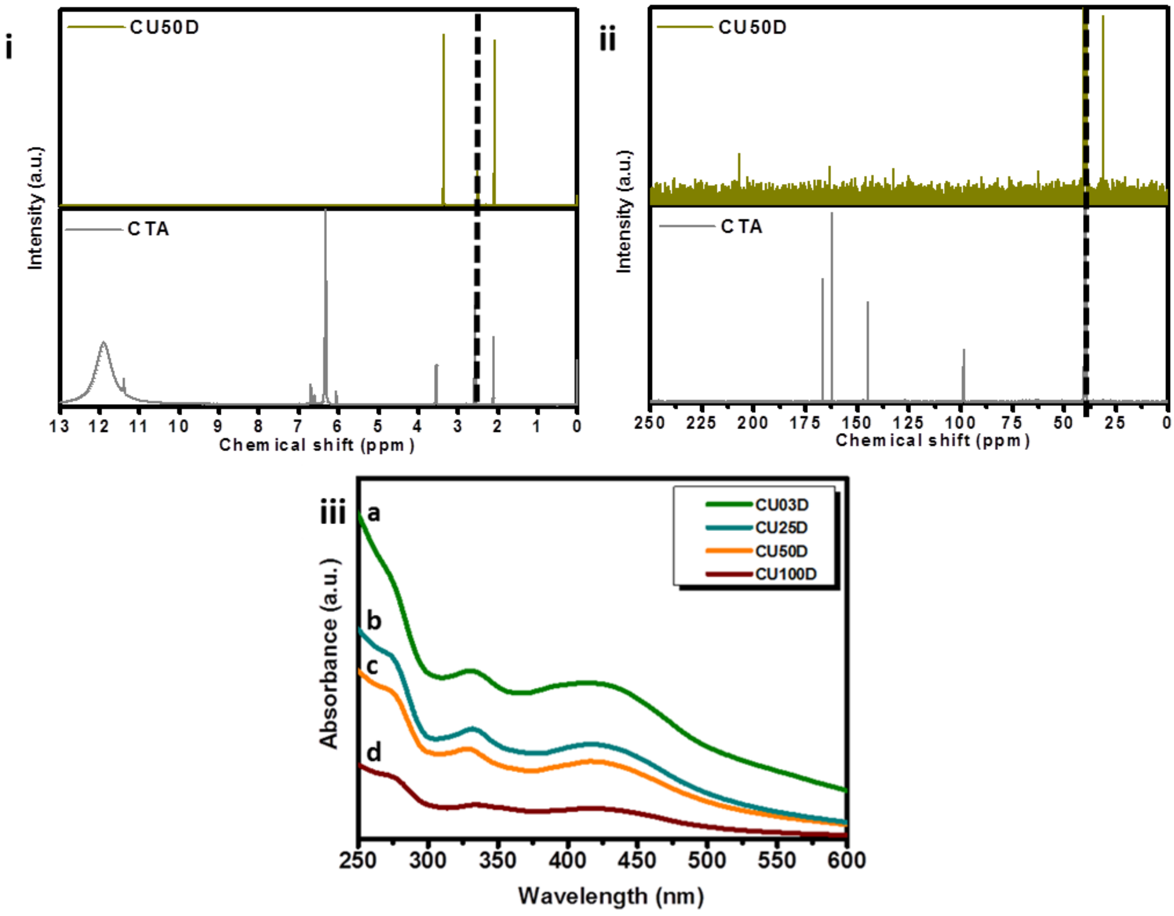

Figure S7. The ^{1}H-NMR (i) and ^{13}C-NMR (ii) spectra of CU50D compared to CTA. The d-DMSO solvent peaks are marked as dashed black line. The UV-Vis spectra (iii) of 0.1 mg/ml aqueous dispersion of CU03D (a), CU25D (b), CU50D (c) and CU100D (d).

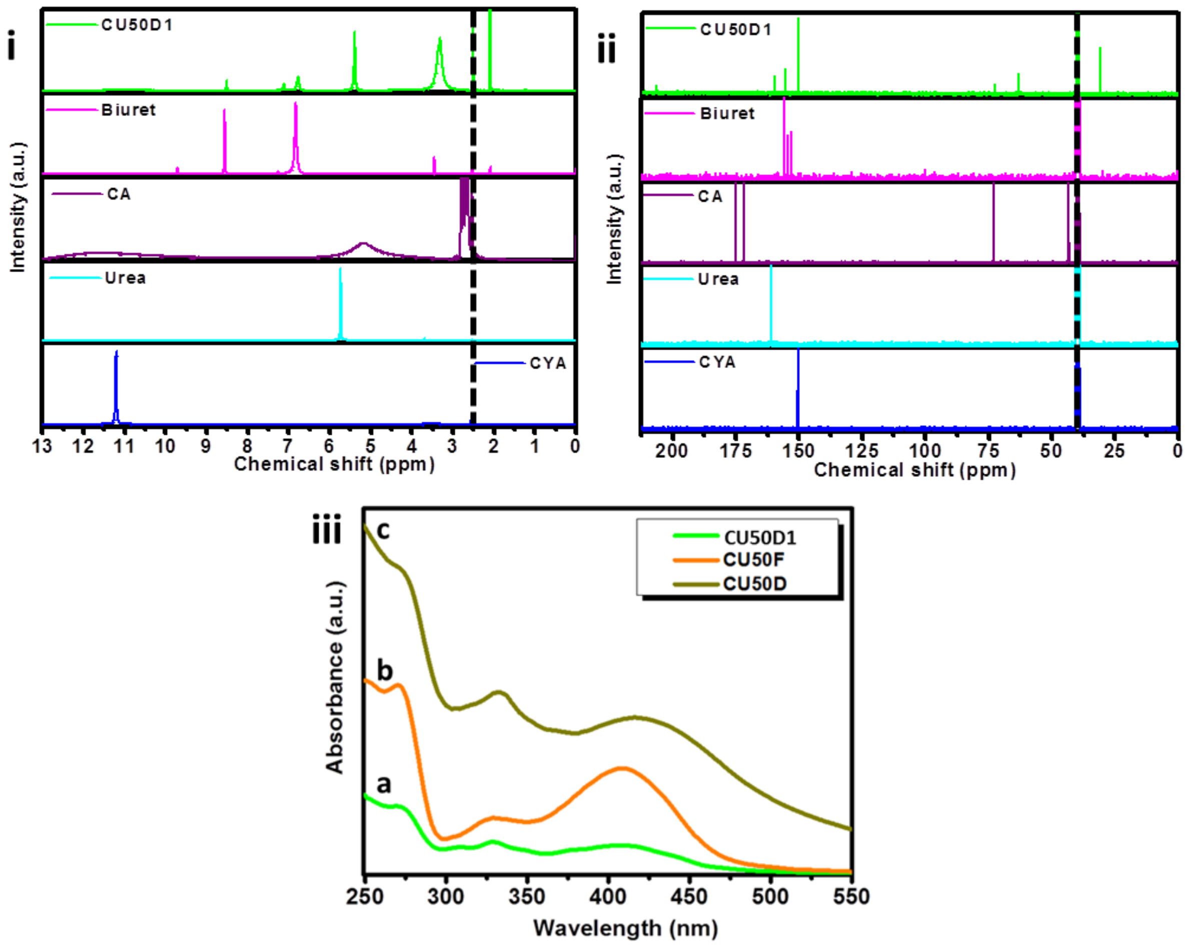

Figure S8. The ^{1}H-NMR (i) and ^{13}C-NMR (ii) spectrum of CU50D1 compared to biuret, CA, urea and CYA. The d-DMSO solvent peaks are marked as dashed black line. The UV-Vis spectra (iii) of aqueous dispersions of CU50D1 (a) compared to CU50F (b) and CU50D (c).

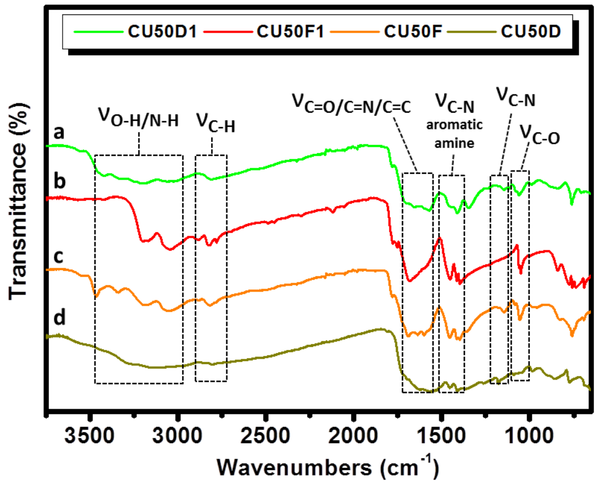

Figure S9. FTIR spectra of CU50D1 (a) compared to CU50F1 (b), CU50F (c) and CU50D (d).

Table S3. Elemental analysis of F-series

Material	F-series			
	C	H	N	O
CU03F	36%	4%	31%	29%
CU25F	31%	4%	35%	30%
CU50F	30%	4%	37%	29%
CU100F	29%	5%	38%	28%

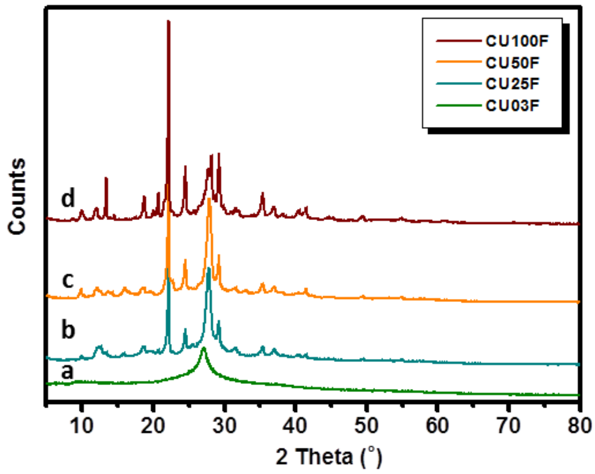

Figure S10. XRD patterns for CU03F (a), CU25F (b), CU50F (c) and CU100F (d).

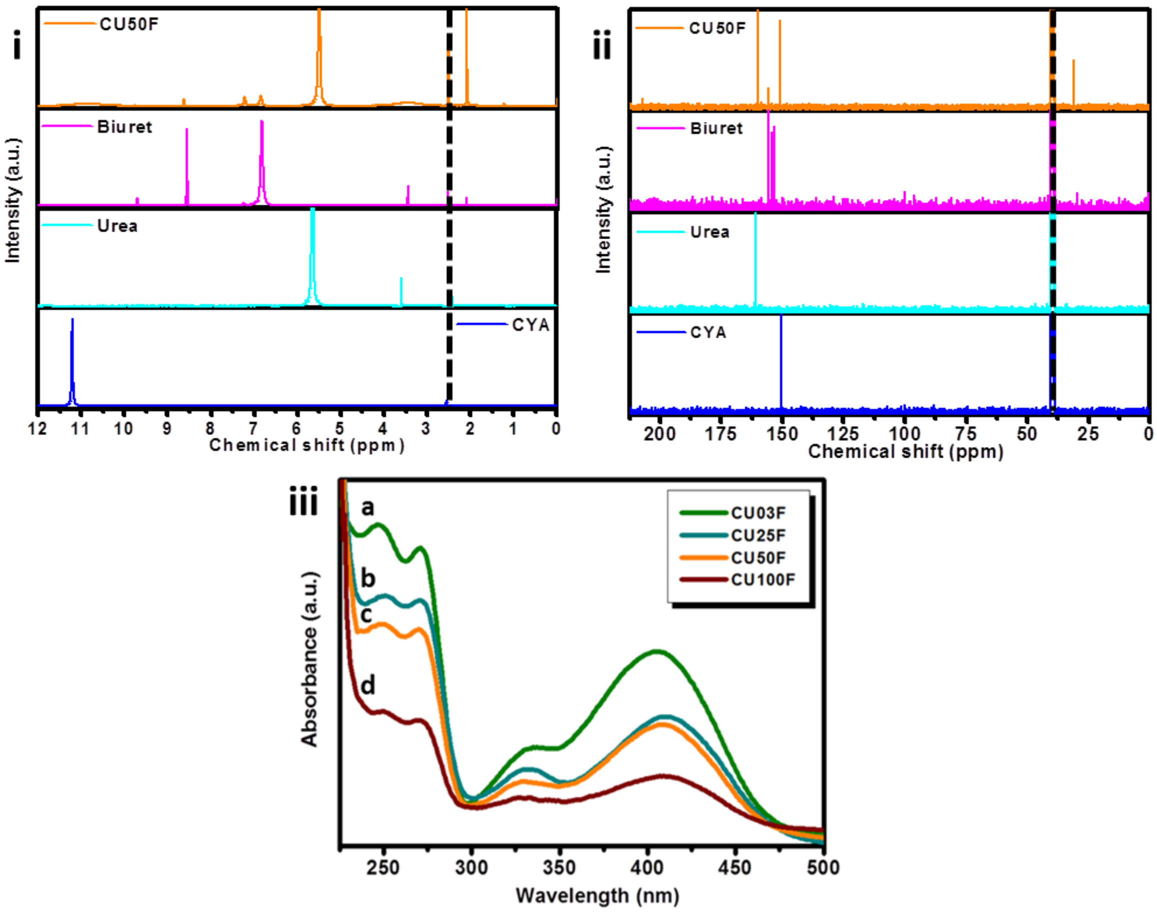

Figure S11. The ^{1}H-NMR (i) and ^{13}C-NMR (ii) spectra of CU50F compared to biuret, urea and CYA. The d-DMSO solvent peaks are marked as dashed black line. The UV-Vis spectra (iii) of 0.1 mg/ml aqueous dispersions of CU03F (a), CU25F (b), CU50F (c), CU100F (d).

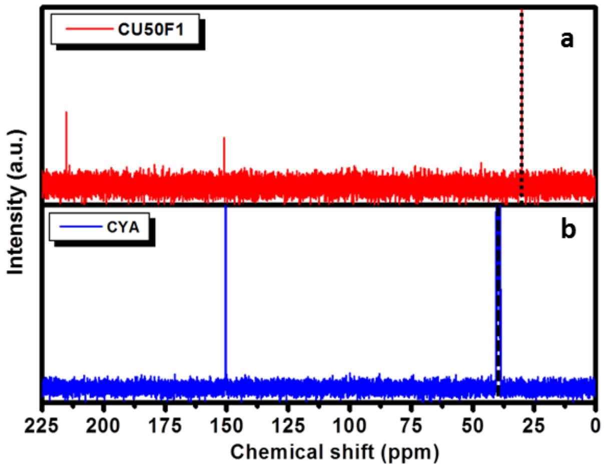

Figure S12. The ^{13}C-NMR spectra of CU50F1 (a) compared to CYA (b). The D_2O (a) and d-DMSO (b) solvent peaks are marked as dashed black line.

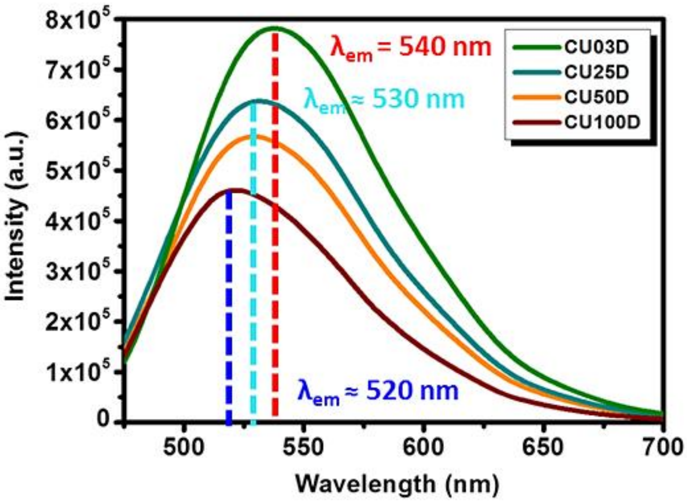

Figure S13. PL spectra of 0.1 mg/ml of CU03D, CU25D, CU50D and CU100D at λ_{ex}= 460 nm.

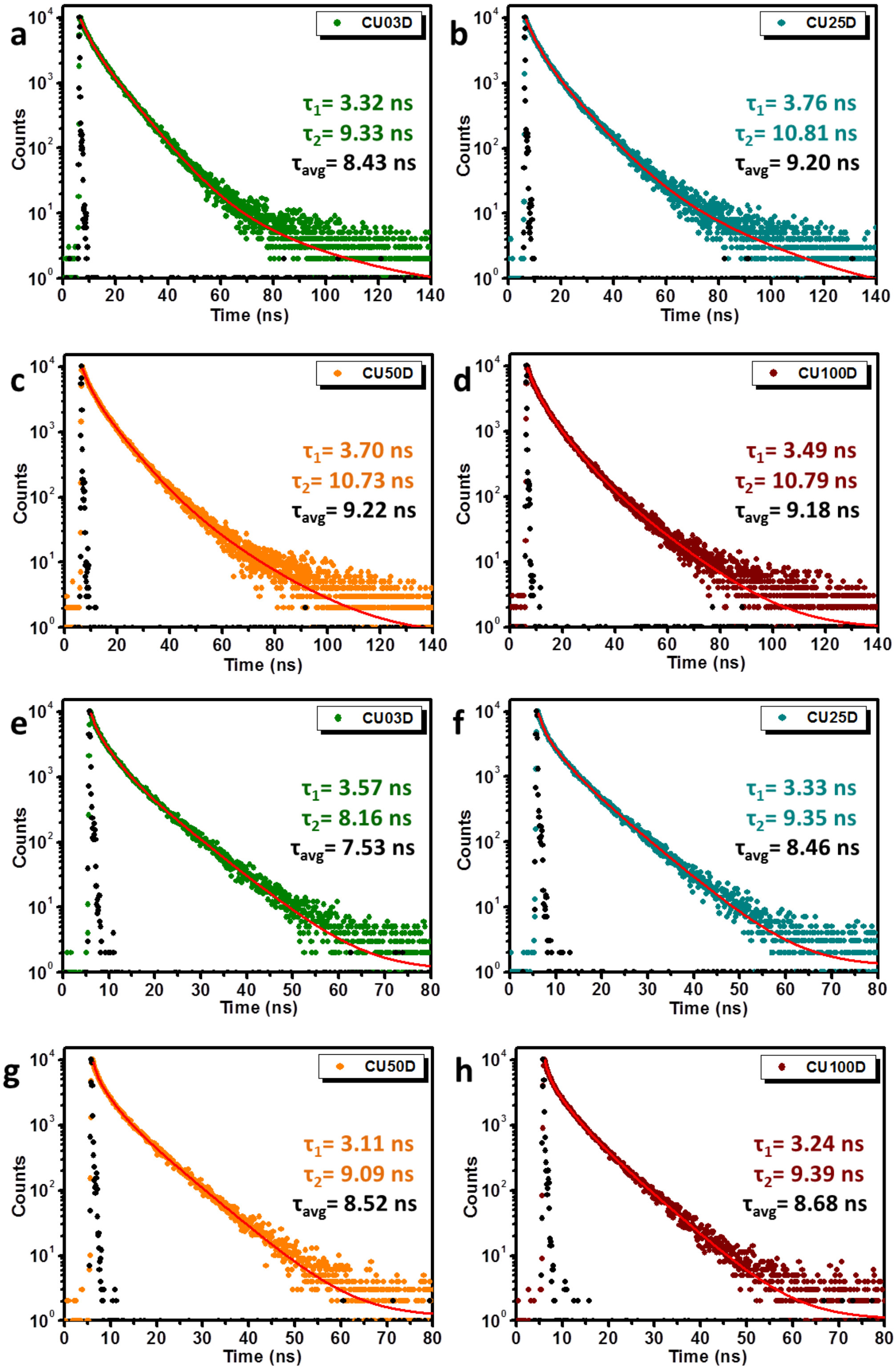

Figure S14. Fluorescence lifetime decays of 0.1 mg /ml aqueous dispersions of CU03D, CU25D, CU50D and CU100D at λ_{ex}= 375 nm (a-d) λ_{ex}= 450 nm (e-h). The points represent the experimental data and the lines correspond to cumulative fitted curves.

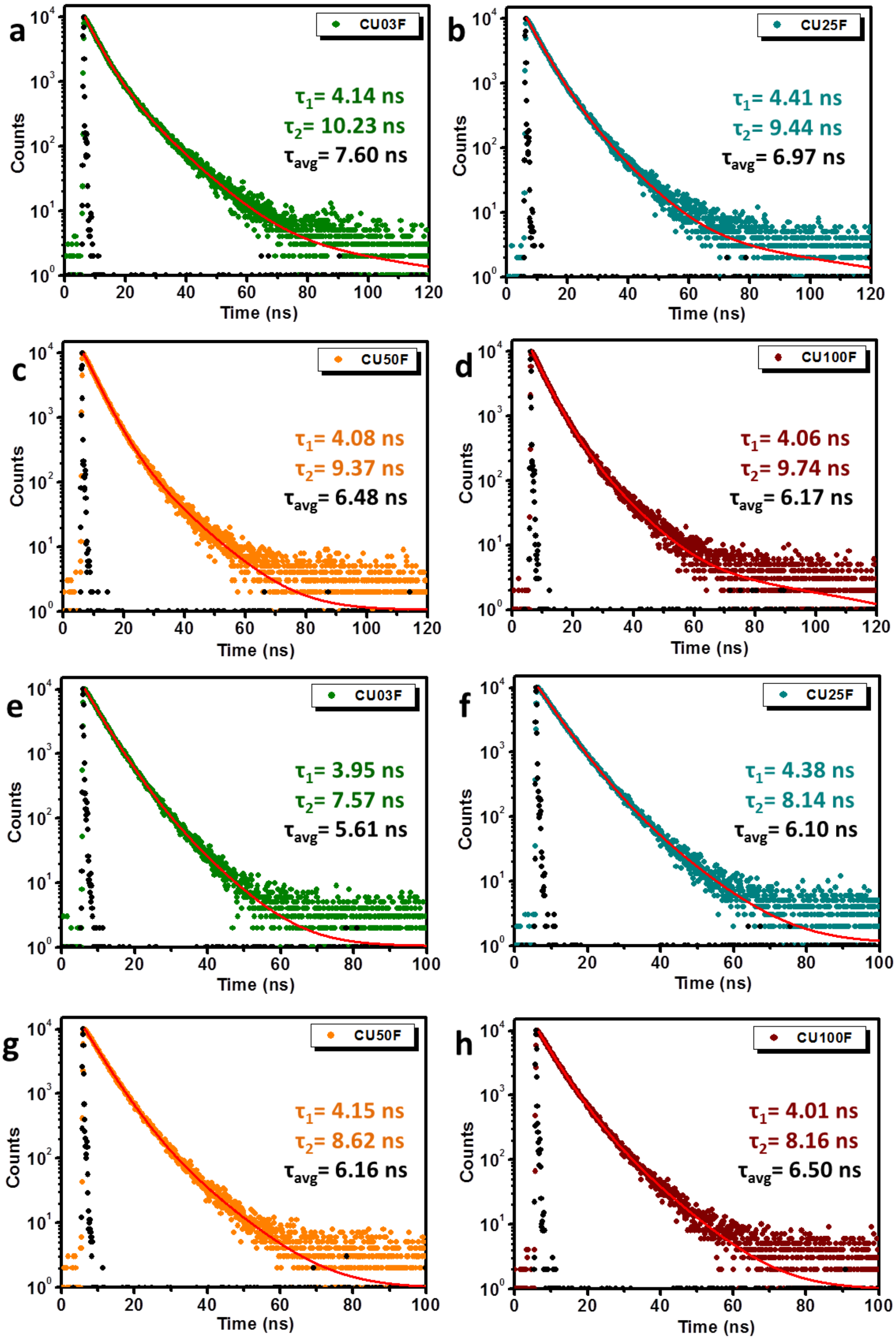

Figure S15. Fluorescence lifetime decays of 0.1 mg /ml aqueous dispersions of CU03F, CU25F, CU50F and CU100F at λ_{ex}= 375 nm (a-d) and λ_{ex}= 450 nm (e-h). The points represent the experimental data and the lines correspond to cumulative fitted curves.

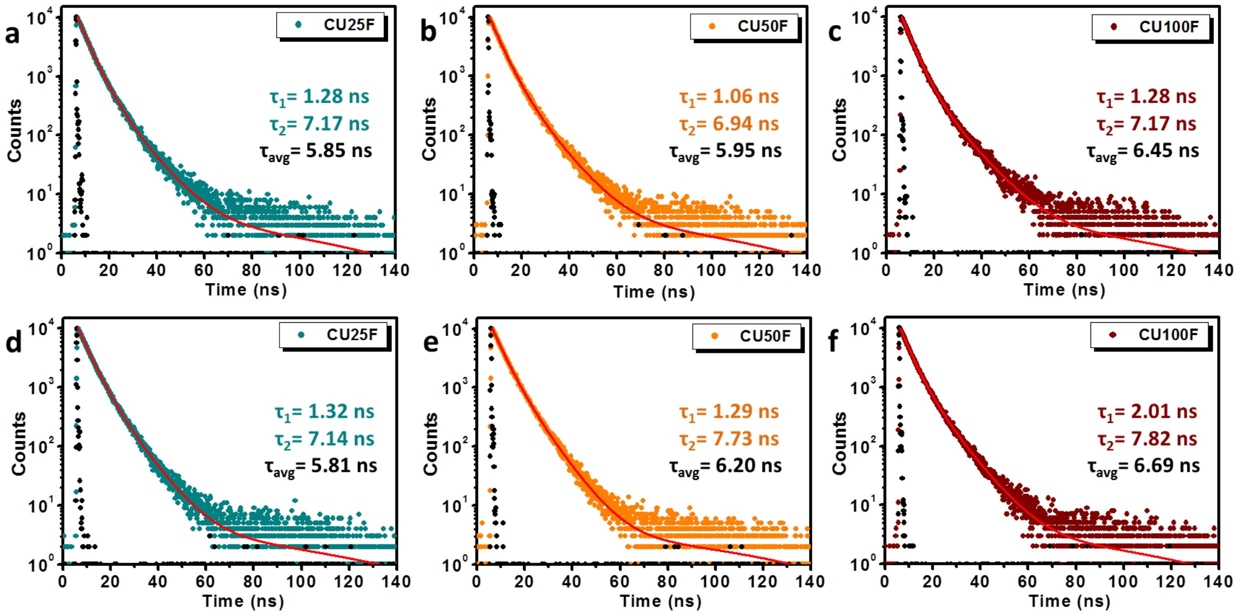

Figure S16. Solid-state PL lifetime decays of CU25F, CU50F and CU100F nanopowders recorded at λ_{ex}= 375nm (a,b,c) and 450 nm (d,e,f). The points represent the experimental data and the lines correspond to the cumulative fitted curves.

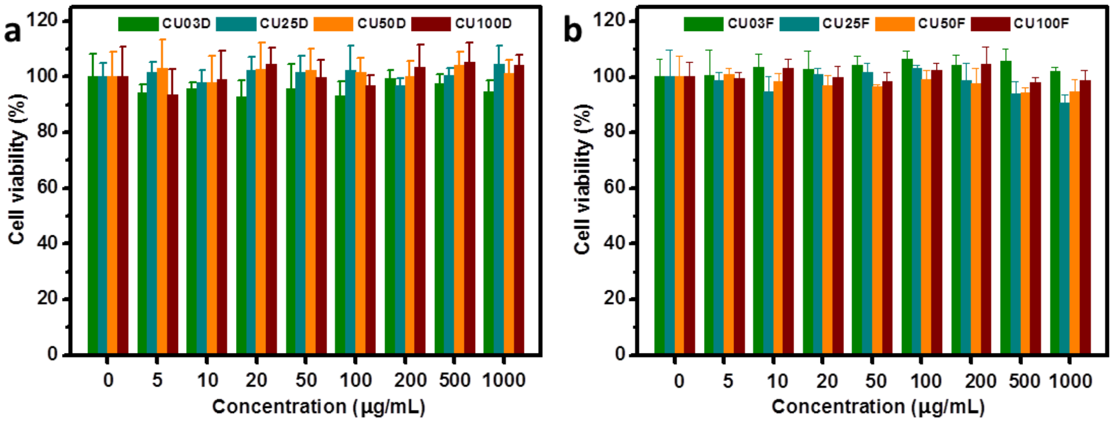

Figure S17. The cell viability of human cancer cervix HeLa cell line calculated from MTT assay after their incubation with D-series (a) and F-series (B) for 24 hours. The data is presented as mean ± SD of triple experiments while percentage of cytotoxicity is represented comparatively to untreated controls.

Table S4. Reduction in bacterial colonies incubated with the D-series and the F-series for 24 hours at 37°C. The data is expressed as mean ± SD of triple experiments while percentage of bacterial colonies decrease is related comparatively to untreated bacteria.

	Escherichia coli		***Staphylococcus aureus***	
**Material**	**% decrease**		**% decrease**	
	**D-series**	**F-series**	**D-series**	**F-series**
**CU03**	**99.0**	**99.9**	**99.9**	**99.9**
**CU25**	**98.2**	**99.9**	**99.9**	**99.9**
**CU50**	**98.6**	**99.6**	**99.7**	**99.9**
**CU100**	**79.0**	**99.5**	**97.2**	**99.2**
